# Structural Insight into Molecular Inhibitory Mechanism of InsP_6_ on African Swine Fever Virus mRNA-Decapping Enzyme g5Rp

**DOI:** 10.1128/jvi.01905-21

**Published:** 2022-04-28

**Authors:** Yan Yang, Changhui Zhang, Xuehui Li, Li Li, Yanjuan Chen, Xin Yang, Yao Zhao, Cheng Chen, Wei Wang, Zhihui Zhong, Cheng Yang, Zhen Huang, Dan Su

**Affiliations:** a State Key Laboratory of Biotherapy and Cancer Center, West China Hospital, Sichuan University and Collaborative Innovation Center of Biotherapy, Chengdu, Sichuan, People’s Republic of China; b Institute of Life Sciences, Chongqing Medical University, Chongqing, People’s Republic of China; c Shanghai Institute for Advanced Immunochemical Studies and School of Life Science and Technology, ShanghaiTech University, Shanghai, China; d School of Life Sciences, Tianjin University, Tianjin, People’s Republic of China; e College of Life Sciences, Sichuan University, Chengdu, China; f Tianjin International Joint Academy of Biotechnology and Medicine, Tianjin, People’s Republic of China; University of Illinois at Urbana Champaign

**Keywords:** g5Rp, ASFV, mRNA-decapping enzyme, Nudix hydrolases, InsP_6_

## Abstract

Removal of 5′ cap on cellular mRNAs by the African swine fever virus (ASFV) decapping enzyme g5R protein (g5Rp) is beneficial to viral gene expression during the early stages of infection. As the only nucleoside diphosphate-linked moiety X (Nudix) decapping enzyme encoded in the ASFV genome, g5Rp works in both the degradation of cellular mRNA and the hydrolyzation of the diphosphoinositol polyphosphates. Here, we report the structures of dimeric g5Rp and its complex with inositol hexakisphosphate (InsP_6_). The two g5Rp protomers interact head to head to form a dimer, and the dimeric interface is formed by extensive polar and nonpolar interactions. Each protomer is composed of a unique N-terminal helical domain and a C-terminal classic Nudix domain. As g5Rp is an mRNA-decapping enzyme, we identified key residues, including K^8^, K^94^, K^95^, K^98^, K^175^, R^221^, and K^243^ located on the substrate RNA binding interfaces of g5Rp which are important to RNA binding and decapping enzyme activity. Furthermore, the g5Rp-mediated mRNA decapping was inhibited by InsP_6_. The g5Rp-InsP_6_ complex structure showed that the InsP_6_ molecules occupy the same regions that primarily mediate g5Rp-RNA interaction, elucidating the roles of InsP_6_ in the regulation of the viral decapping activity of g5Rp in mRNA degradation. Collectively, these results provide the structural basis of interaction between RNA and g5Rp and highlight the inhibitory mechanism of InsP_6_ on mRNA decapping by g5Rp.

**IMPORTANCE** ASF is a highly contagious hemorrhagic viral disease in domestic pigs which causes high mortality. Currently, there are still no effective vaccines or specific drugs available against this particular virus. The protein g5Rp is the only viral mRNA-decapping enzyme, playing an essential role in the machinery assembly of mRNA regulation and translation initiation. In this study, we solved the crystal structures of g5Rp dimer and complex with InsP_6_. Structure-based mutagenesis studies revealed critical residues involved in a candidate RNA binding region, which also play pivotal roles in complex with InsP_6_. Notably, InsP_6_ can inhibit g5Rp activity by competitively blocking the binding of substrate mRNA to the enzyme. Our structure-function studies provide the basis for potential anti-ASFV inhibitor designs targeting the critical enzyme.

## INTRODUCTION

African swine fever virus (ASFV), which is an enveloped double-stranded DNA virus, has a genome that varies between 170 and 193 kbp with 151 to 167 open reading frames depending on the virus strain (1, 2). As the only known DNA arbovirus, ASFV is the sole member of the *Asfarviridae*, a family of African swine fever-like viruses that are relatively independent of the host cell transcriptional machinery for viral replication (3, 4). The ASFV infection of domestic swine can result in various disease forms, ranging from highly lethal to subclinical depending on the contributing viral and host factors (5). Since 2018, ASFV has spread into China and led to a high mortality rate in domestic pigs (6, 7). Currently, there are still no effective vaccines or specific drugs available against this particular virus (8, 9).

During an ASFV infection, protein synthesis in the host cell is inhibited as a result of a massive degradation of host cellular mRNAs in the cytoplasm of infected cells (10, 11). As part of its strategy to inhibit host cellular translation and promote viral protein synthesis instead, the virus targets the mRNAs of the host cell using specific enzymes (12). Hydrolysis of the 5′ cap structure (m^7^GpppN) on eukaryotic mRNAs, a process known as decapping, is considered to be a crucial and highly regulated step in the degradation of mRNA (13). Some viruses including ASFV and vaccinia virus (VACV) can harbor decapping enzymes for control of viral and cellular gene expression (14). Two poxvirus Nudix hydrolases, D9 and D10, have been confirmed with intrinsic mRNA-decapping activity, although the two decapping enzymes appear to have some differences in substrate recognition (15, 16).

Nudix hydrolases (nucleoside diphosphate-linked moiety X) are widely present in bacteria, archaea, and eukarya, where they belong to a superfamily of hydrolytic enzymes that catalyze the cleavage of nucleoside diphosphates and the decapping of the 5′ cap of mRNAs, the latter of which plays a pivotal role in mRNA metabolism (17, 18). Mammalian cells have about 30 different genes with Nudix motifs, including Dcp2, Nudt16, and NUDT3/DIPP1, which cleaves mRNA caps in mRNA degradation by the 5′-3′ decay pathway *in vivo* (19–21). The mRNA-decapping enzyme g5R protein (g5Rp), which is the only Nudix hydrolase in ASFV, shares sequence similarity to the mRNA-decapping enzymes Dcp2 in Schizosaccharomyces pombe and D9 or D10 in VACV (22–24). However, g5Rp and its Nudix homologs D9 and D10 exhibit higher hydrolytic activity toward diphosphoinositol polyphosphates and dinucleotide polyphosphates than toward cap analogs (25, 26). Similar to Dcp2, these Nudix hydrolases cleave the mRNA cap attached to an RNA moiety, predicating that RNA binding is crucial for performing its mRNA-decapping activity (16). Recently, structural study has confirmed that the Nudix protein CFI_m_25 has a sequence-specific RNA binding capability (27). The requirement of RNA binding for the majority of the Nudix decapping enzymes suggest that the members of the Nudix family also belong to RNA binding proteins.

The viral mRNA-decapping enzyme g5Rp is expressed in the endoplasmic reticulum from the early stage of ASFV infection and accumulates throughout the infection process, playing an essential role in the machinery assembly of mRNA regulation and translation initiation (23). Like other members of the Nudix family, g5Rp has a broader range of nucleotide substrate specificity, including that for a variety of guanine and adenine nucleotides and dinucleotide polyphosphates (25). Generally, g5Rp has two distinct enzymatic activities *in vitro* (*viz.*, diphosphoinositol polyphosphate hydrolase activity and mRNA-decapping activity), implying that it plays roles in viral membrane morphogenesis and mRNA regulation during viral infections (28). In light of these biochemical observations, the elucidation of the structure of g5Rp is of fundamental importance for our understanding of the molecular mechanisms through which it degrades cellular RNAs and regulates viral gene expression.

Here, we report the crystal structure of g5Rp and its complex structure with InsP_6_. Combined with biochemical experiments, the dimeric form of g5Rp and three RNA binding surfaces on each protomer are critical to substrate RNA binding of g5Rp. The g5Rp-InsP_6_ complex structure shows that two of the RNA binding surfaces are occupied by InsP_6_, indicating that InsP_6_ may play a role in its ability to inhibit g5Rp-RNA binding activity. Meanwhile, we evaluate the inhibitor effect of InsP_6_ on the mRNA-decapping enzyme activity of g5Rp. Therefore, we proposed that such inhibition could be caused by the competition of InsP_6_ with substrate mRNA for binding to g5Rp. Furthermore, we show in detail how InsP_6_ inhibits g5Rp activity by occupying the RNA binding interfaces on g5Rp, thereby competitively blocking the binding of substrate mRNA to the enzyme. These results suggest InsP_6_ or its structural analogs may be involved in the manipulation of the mRNA-decapping process during viral infections and provide an essential structural basis for the development of ASFV chemotherapies in the future.

## RESULTS

### Characterization of recombinant ASFV g5Rp.

**FIG 1:**
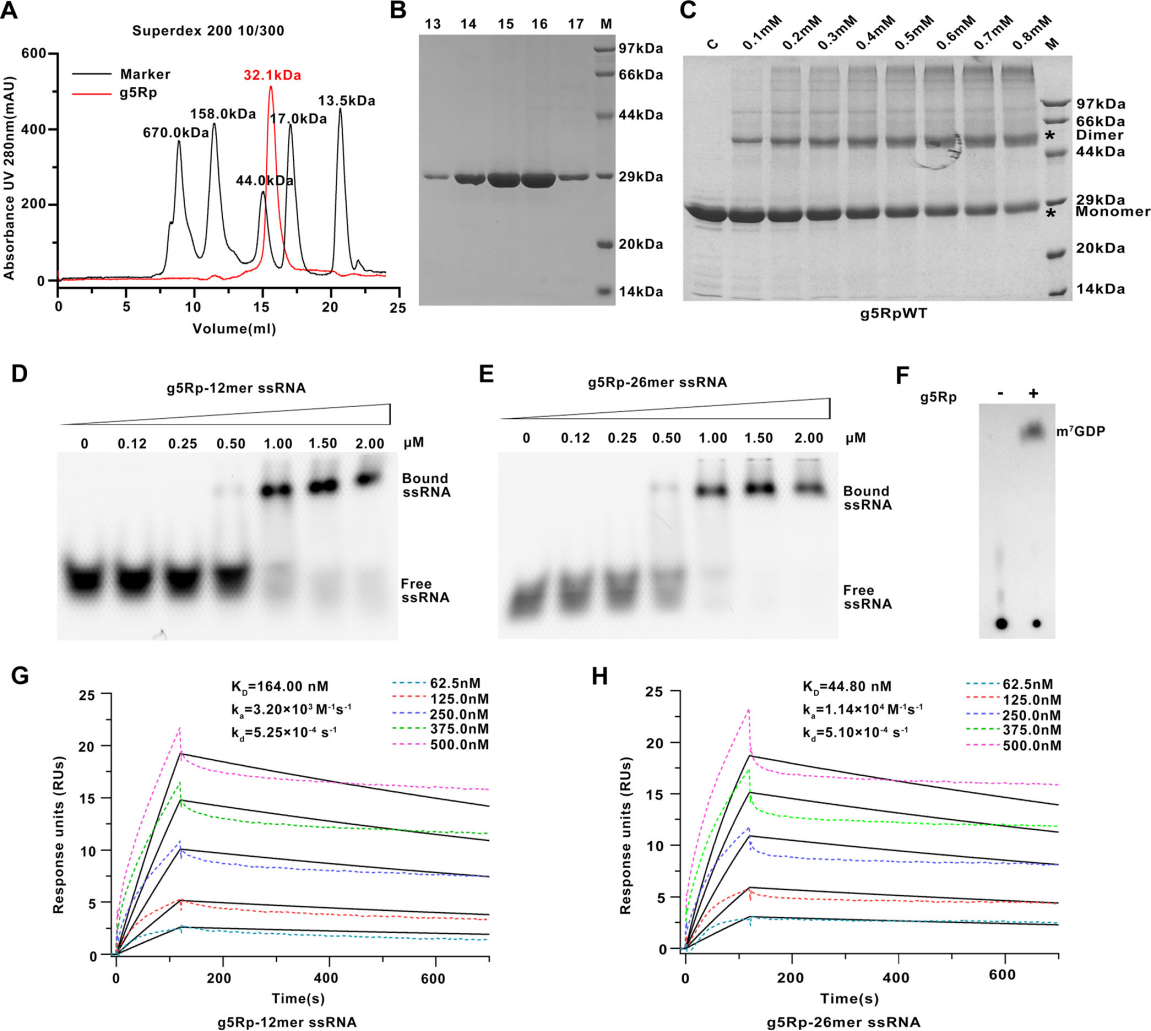
Characterization of the African swine fever virus decapping enzyme g5Rp. (A) The elution profile of g5Rp in a Superdex 200 10/300 column and the molecular weight of the standards and g5Rp are shown in this picture. (B) SDS-PAGE result for g5Rp. M is the protein marker. (C) The cross-linking of g5Rp by using chemical reagent EGS. The different concentrations of EGS with g5Rp in the reaction system are indicated above the gel. (D and E) The binding abilities of g5Rp to 12-mer and 26-mer ssRNAs were determined by EMSA; in the reaction, the protein concentration is marked on the figure, and the nucleic acid concentration is 0.25 μM. (F) The decapping activity of g5Rp. (G and H) The binding abilities of g5Rp to 12-mer and 26-mer ssRNAs were determined by surface plasmon resonance. SPR data were analyzed using a 1:1 binding model, and black lines represent curve fits.

Recombinant wild-type (WT) ASFV g5Rp (residues 1 to 250) was expressed in Escherichia coli with an N-terminal His_6_ tag. The purified g5Rp was eluted from a Superdex 200 column (GE Healthcare) with a major elution volume of 15.6 mL, indicating an approximate molecular weight of 32.1 kDa (Fig. 1A). The fractions were further analyzed by sodium dodecyl sulfate-polyacrylamide gel electrophoresis (SDS-PAGE), showing a g5Rp band of 29.9 kDa (Fig. 1B). A cross-linking assay confirmed that g5Rp exists as a stable homodimer in solution (Fig. 1C).

We first characterized the nucleic acid binding ability of g5Rp with different lengths of single-stranded RNA (12-mer and 26-mer ssRNA). Electrophoretic mobility shift assay (EMSA) results demonstrated that g5Rp binds ssRNA (0.25 μM) at the lowest concentration of 0.5 μM (Fig. 1D and E). Furthermore, we measured the binding affinity of wild-type (WT) g5Rp for ssRNA by using surface plasmon resonance (SPR) (Fig. 1G and H). The enzyme exhibited a stronger binding affinity to ssRNAs with the following equilibrium dissociation constants: 12-mer *K_D_* = 164.0 nM and 26-mer *K_D_* = 44.8 nM. The kinetic analysis of the binding experiments is shown in Table 1. These results indicate that g5Rp possesses a higher affinity with long ssRNA. Next, we reevaluated the decapping activity of recombinant g5Rp by incubating the protein with a ^32^P-cap-labeled RNA substrate in a reaction. The products of the reaction were resolved by polyethyleneimine (PEI)-cellulose thin-layer chromatography (TLC) and detected by autoradiography (23). As shown in Fig. 1F, the recombinant g5Rp in the decapping reaction released 7-methylguanosine cap (m^7^GDP) product efficiently. In contrast, the ^32^P-cap-labeled RNA substrate as control remained at the origin of the plate. These results suggest that the recombinant g5Rp possesses efficient mRNA-decapping enzyme activity.

**TABLE 1:** Kinetic analysis of SPR

Ligand	Analyte	*K_D_*	*k_a_* (1/Ms)	*k_d_* (1/s)	*R*_max_ (RU)	Chi-square (RU^2^）
26-mer RNA	g5Rp-WT	4.48E−08	1.14e+04	5.10e−04	38.8	2.00
		3.81E−08	1.61e+04	6.12e−04	33.5	3.92
	G5Rp-ΔC	5.07E−08	1.85e+04	9.38e−04	63.2	10.4
		4.70E−08	1.47e+04	6.92e−04	46.1	4.04
	G5Rp-I84A/I116A/	2.24E−07	2.07e+03	4.65e−04	1,142.7	1,930
	L200A/I206A/F206A	5.75E−07	1.05e+03	6.05e−04	1,013.0	1,470

12-mer RNA	g5Rp-WT	1.64E−07	3.20e+03	5.25e−04	112.9	1.23
		1.15E−07	5.33e+03	6.13e−04	77.8	4.51
	G5Rp-ΔC	3.90E−08	2.12e+04	8.28e−04	62.3	11.6
		2.87E−08	2.84e+04	8.14e−04	46.0	5.20
	G5Rp-I84A/I116A/	1.15E−07	2.61e+03	3.01e−04	435.1	324
	L200A/I206A/F206A	2.25E−07	2.52e+03	5.68e−04	348.2	136

### Overview of the ASFV g5Rp structure.

**FIG 2:**
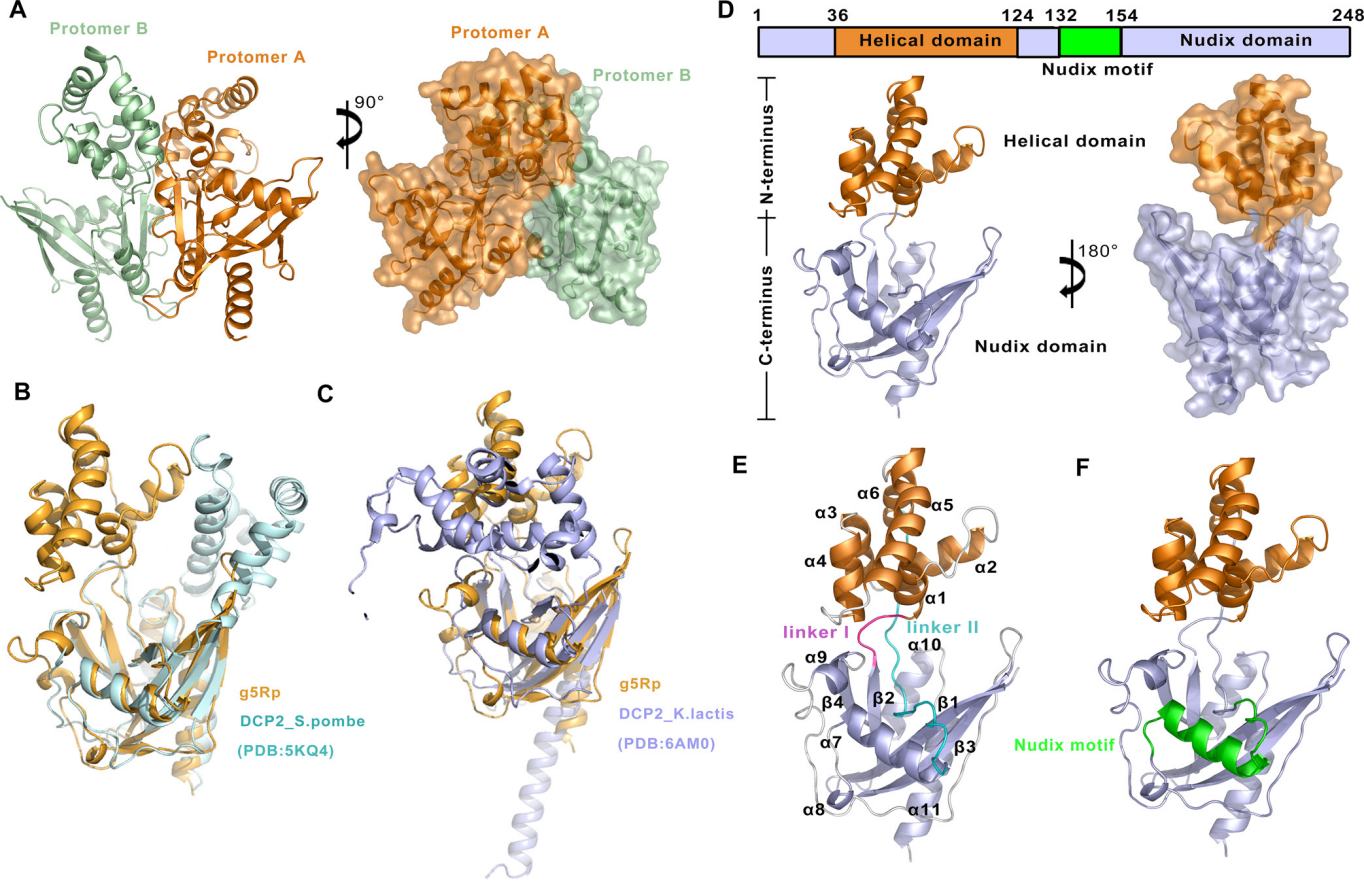
Structure of the African swine fever virus decapping enzyme g5Rp. (A) The dimeric structure of the African swine fever virus decapping enzyme g5Rp. The dimer consists of protomers A and B in a back-to-back orientation. Protomer A is colored orange, and protomer B is colored pale green. (B and C) Superposition of g5Rp Nudix domain with DCP2 from S. pombe (mRNA-decapping complex subunit 2, PDB accession no. 5KQ4) and DCP2 from Kluyveromyces lactis (enhancer of mRNA-decapping protein 3, PDB accession no. 6AM0). g5Rp from ASFV is shown in bright orange, 5KQ4 is shown in pale cyan, and 6AM0 is shown in light blue. (D) “Boxing glove” arrangement of the overall structure of g5Rp. The division of the domain is based on the g5Rp structure, where each domain is color coded, with the helical domain in orange and the Nudix domain in light blue (containing the Nudix motif, in green). The N terminus and C terminus domains are marked by the black box. (E) The structural information of g5Rp. The 11 α-helices, 4 β-sheets, and loops are indicated in the structure. The loops are colored in white, except for linker I (in magenta) and linker II (in cyan). (F) The details of the Nudix motif in g5Rp. The Nudix motif is colored in green.

To investigate structural insights into the catalytic mechanism of g5Rp, we determined its dimeric structure by single-wavelength anomalous diffraction (SAD) phases using selenomethionine (SeMet)-labeled protein. As shown in Fig. 2A, the g5Rp dimer is composed of two protomers that each adopt a “boxing glove” shape with a distinct helical domain and Nudix domain (Fig. 2D). The helical domain (residues 36 to 124) forms a globin-fold-like feature composed of six α-helices (α1 to α6) that connects to the Nudix domain by two hinge linkers (linker I, residues 32 to 35; linker II, residues 119 to 139). The Nudix domain (residues 1 to 35 and 125 to 250) consists of a central curved β-sheet (β1, β2, β3, β4) surrounded by five α-helices (α7 to α11) and several loops, thereby forming a classic α-β-α sandwich structure. Linker II splits the top of the β-sheet to connect α6 and α7 (Fig. 2E). The Nudix motif located in the center of the Nudix domain is highly conserved and comprises the loop-helix-loop architecture that contains the Nudix signature sequence extending from residues ^132^GKPKEDESDLTCAIREFEEETGI^154^ in g5Rp (Fig. 2F). The sequence of the g5Rp Nudix motif matches the classic pattern of the Nudix motif in the Nudix hydrolase superfamily, that is, GX_5_EX_7_REUXEEXGU, where X is any residue and U is Ile, Leu, or Val (29, 30). Using the Dali server (31), we compared the structure of g5Rp with that of other proteins in the Protein Data Bank (PDB), whereupon 46 structures were found to be likely homologous to the enzyme, with Z-scores in the range of 8 to 20 (data not shown). However, all the listed protein structures shared high architectural similarity only with the Nudix domain located in the C terminus of g5Rp. Therefore, a search on the Dali server was carried out for the helical domain alone, whereupon no homologous structure with a Z-score above 4 was found, suggesting that the helical domain of g5Rp adopts a novel fold. Compared with the structures of Dcp2 in a number of different conformations, g5Rp shows a unique globin-fold-like domain (Fig. 2B and C).

**FIG 3:**
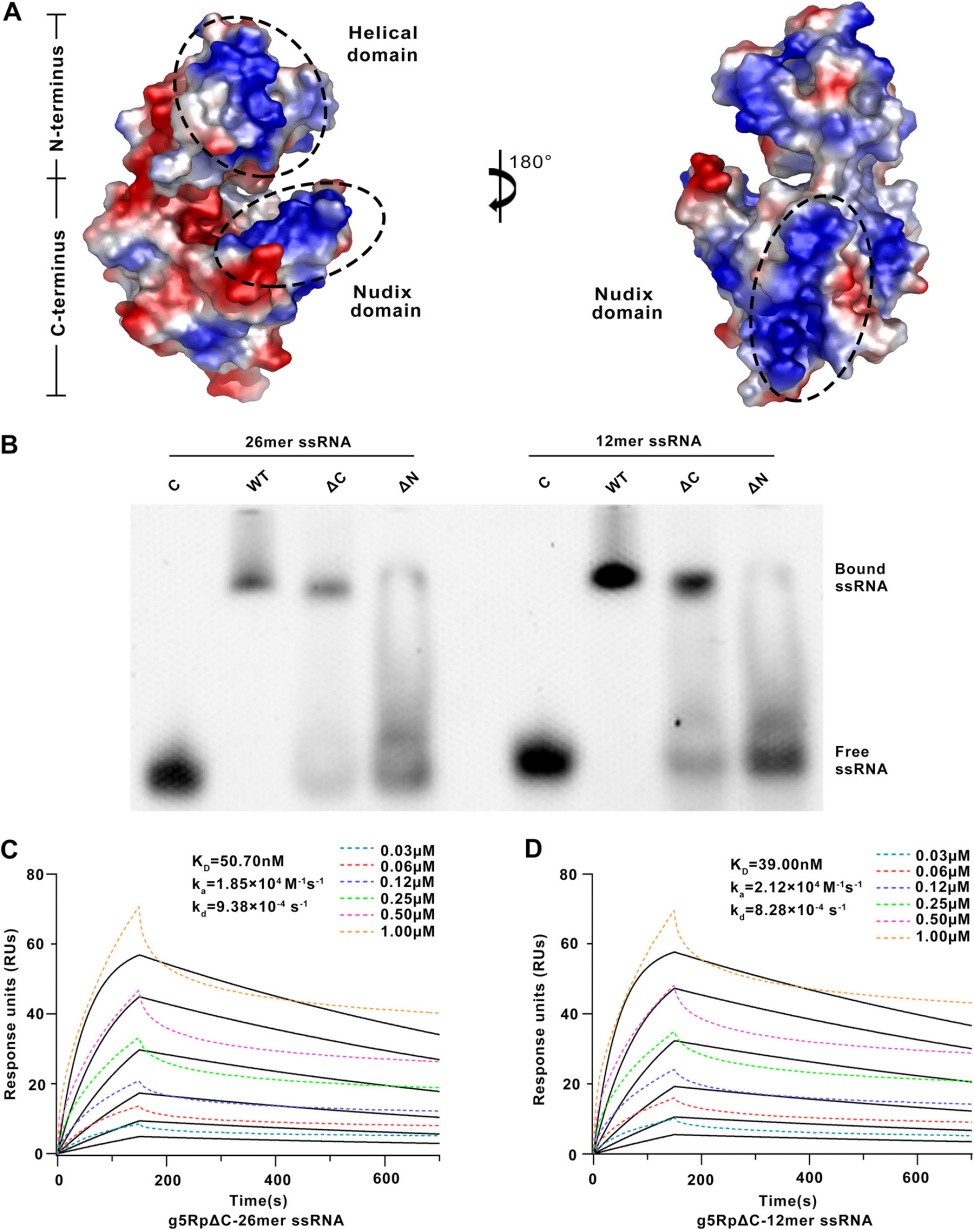
Surface charge distribution of g5Rp and the biochemical information of the African swine fever virus decapping enzyme truncated g5Rp. (A) Surface charge distribution of g5Rp. The range of electrostatic surface potential is shown from −74.712 kT/e in red to +74.712 kT/e in blue. The three highly positively charged areas are marked by the black dashed lines. (B) The binding abilities of truncated g5Rp (the final concentrations of WT and ΔC are 2.00 μM while that of ΔN is 50 μM) to 26- and 12-mer ssRNAs (final concentration, 0.25 μM) were determined by EMSA. (C and D) The binding abilities of truncated g5Rp ΔC to 26- and 12-mer ssRNAs were determined by SPR. SPR data were analyzed using a 1:1 binding model, and black lines represent curve fits.

A previous study showed that the helical domain of g5Rp is the major mediator of RNA interaction (28). However, the positively charged surface of the g5Rp structure overlaps both the helical domain and the Nudix domain that may exhibit RNA binding activity (Fig. 3A). We proposed that both positively charged regions could contribute to g5Rp-RNA interaction. To test the hypothesis, we measured the binding of the truncation variants g5RpΔC (helical domain, residues 36 to 124) and g5RpΔN (Nudix domain, connecting residues 1 to 35 and 125 to 250 directly) to ssRNAs (12-mer and 26-mer), respectively. Our EMSA results showed that both the helical domain (g5RpΔC) and Nudix domain (g5RpΔN) of g5Rp are involved in ssRNA interaction (Fig. 3B). The helical domain exhibited *K_D_* values of 39.0 and 50.7 nM for the surface-immobilized 12- and 26-mer ssRNAs measured by SPR, respectively (Fig. 3C and D). In contrast, the *K_D_* values of wild-type g5Rp for the ssRNAs (12-mer *K_D_* = 164.0 nM, 26-mer *K_D_* = 44.8 nM) are slightly lower than that of the helical domain with ssRNAs, indicating that both full-length and truncated g5Rp associated with RNA with high affinity.

### The dimeric structure of g5Rp.

**FIG 4:**
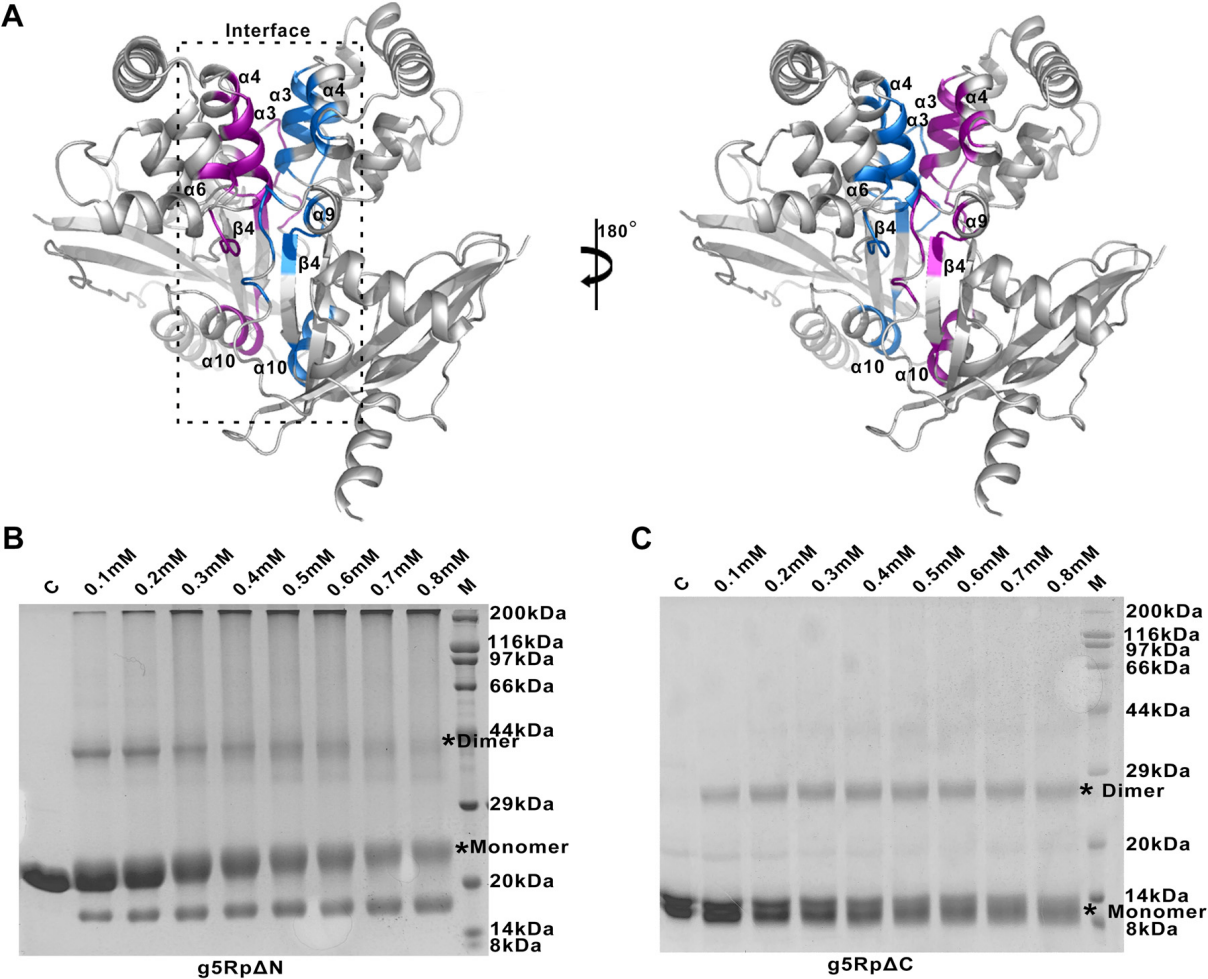
The detailed structural information of dimeric g5Rp and the cross-linking result of truncated g5Rp. (A) The detailed structural information of dimeric g5Rp. The residues of protomers A and B involved in the interface are colored purple and blue, respectively. (B and C) The cross-linking of truncated g5Rp by EGS. The different concentrations of EGS with g5Rp in the reaction system are indicated above the gel.

**FIG 5:**
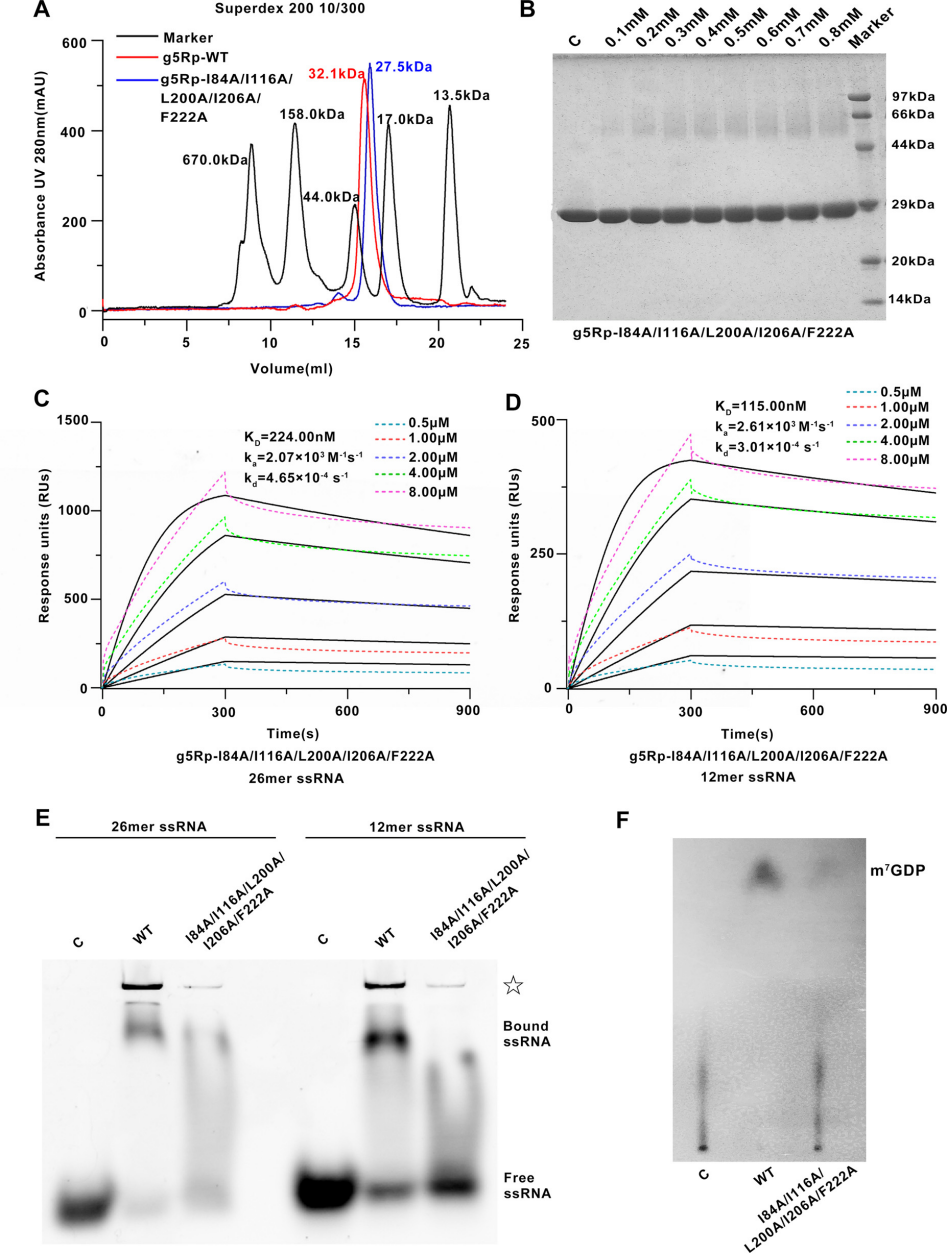
The biochemical information of the African swine fever virus decapping enzyme monomeric g5Rp. (A) The elution profile of g5Rp-WT and g5Rp-I84A/I116A/L200A/I206A/F222A in a Superdex 200 10/300 column; the molecular weight of the standards and g5Rp is shown in this picture. (B) The cross-linking of g5Rp-I84A/I116A/L200A/I206A/F222A by EGS. The different concentrations of EGS with mutants in the reaction system are indicated above the gel. (C and D) The binding abilities of monomeric g5Rp (I84A/I116A/L200A/I206A/F222A) to 26- and 12-mer ssRNAs were determined by SPR. SPR data were analyzed using a 1:1 binding model, and black lines represent curve fits. (E) The binding abilities of monomeric g5Rp (the final concentrations of WT and I84A/I116A/L200A/I206A/F222A are 2.00 μM) to 26- and 12-mer ssRNAs (final concentration, 0.25 μM) were determined by EMSA. The white star represents the complex precipitation in the gel. (F) The decapping activities of g5Rp-I84A/I116A/L200A/I206A/F222A.

When recombinant g5Rp was subjected to gel filtration chromatography to estimate molecular weight, it migrated as a single population of molecules at a molecular mass consistent with a monomer. However, g5Rp dimerization was consistent with cross-linking experiments (Fig. 1A and C). To obtain more information about the interfaces and likely biological assemblies of g5Rp, we analyzed its structure using the PDB-related interactive tool Proteins, Interfaces, Structures and Assemblies (PDBePISA) (32). The results suggested that g5Rp forms a stable symmetric dimer in crystal packing. The dimer was composed of two protomers (A and B) positioned in an orientation similar to two boxing gloves stuck together back to back (Fig. 2A). The dimer interfaces were stabilized mainly by hydrophobic interactions. Furthermore, a network of hydrogen bonds conferred additional stability on the interface. One interface was composed of four α-helices (α3 and α4 from each A and B protomer) from the N terminus of each protomer. Residues Ile^64^, Asn^65^, Arg^67^, Leu^68^, Leu^69^, Lys^71^, Thr^72^, Arg^77^, Tyr^80^, His^81^, and Ile^84^ located in helices α3 and α4 played pivotal roles in stabilizing the dimeric form of the protein. The other dimer interface was located at the linker II portion of the C terminus and one solvent contact surface of the Nudix domain. Residues Ile^116^, Asn^117^, Ala^119^, Lys^120^, Gly^121^, Ser^122^, Gly^123^, and Thr^124^ located on linker II and residues Asn^195^, Met^196^, Leu^198^, Ser^199^, Leu^200^, Gln^201^, Ile^206^, Ser^210^, Lys^211^, Gln^215^, Glu^218^ and Ala^219^ at the Nudix domain formed hydrogen bonds in the dimer interface, with further contributions from a hydrophobic patch composed of Ile^206^, Ile^209^, Phe^222^, and Ile^223^ (Fig. 4A). To determine the multimeric state of g5Rp in solution and to examine which of its termini is critical for its dimerization, we measured the multimerization of two g5Rp truncation variants (g5RpΔN and g5RpΔC) using cross-linking experiments. The results showed that the wild type, N terminus, and C terminus of g5Rp all formed a dimeric conformation in solution (Fig. 4B and C). The g5Rp mutant I84A/I116A/L200A/I206A/F222A that prepared to dissociate the dimeric form of g5Rp was successful in altering a monomeric state, even the dimeric total buried area of 3,050 Å^2^. Wild-type g5Rp and mutants were subjected to gel filtration chromatography, showing that the mutant I84A/I116A/L200A/I206A/F222A has a larger retention volume, corresponding to a lower molecular weight (Fig. 5A). The protein cross-linking experiment showed that the dimeric conformation was significantly reduced in solution for the mutant (Fig. 5B). The ssRNA binding ability of the monomeric mutant has been measured by SPR and EMSA. The monomeric mutant with analyte concentrations was passed over immobilized ssRNA. The resultant sensorgrams are shown in Fig. 5C and D, and kinetic analysis is shown in Table 1. EMSA data are shown in Fig. 5E. Both measurements produced consistent results indicating that the g5Rp mutant I84A/I116A/L200A/I206A/F222A partially impaired the RNA binding ability. Therefore, we proposed that the dimeric g5Rp is preferred for efficient RNA binding. Meanwhile, mRNA-decapping assays showed that the decapping activity of mutant I84A/I116A/L200A/I206A/F222A dropped greatly (Fig. 5F).

### Structure of the g5Rp-InsP_6_ complex.

**FIG 6:**
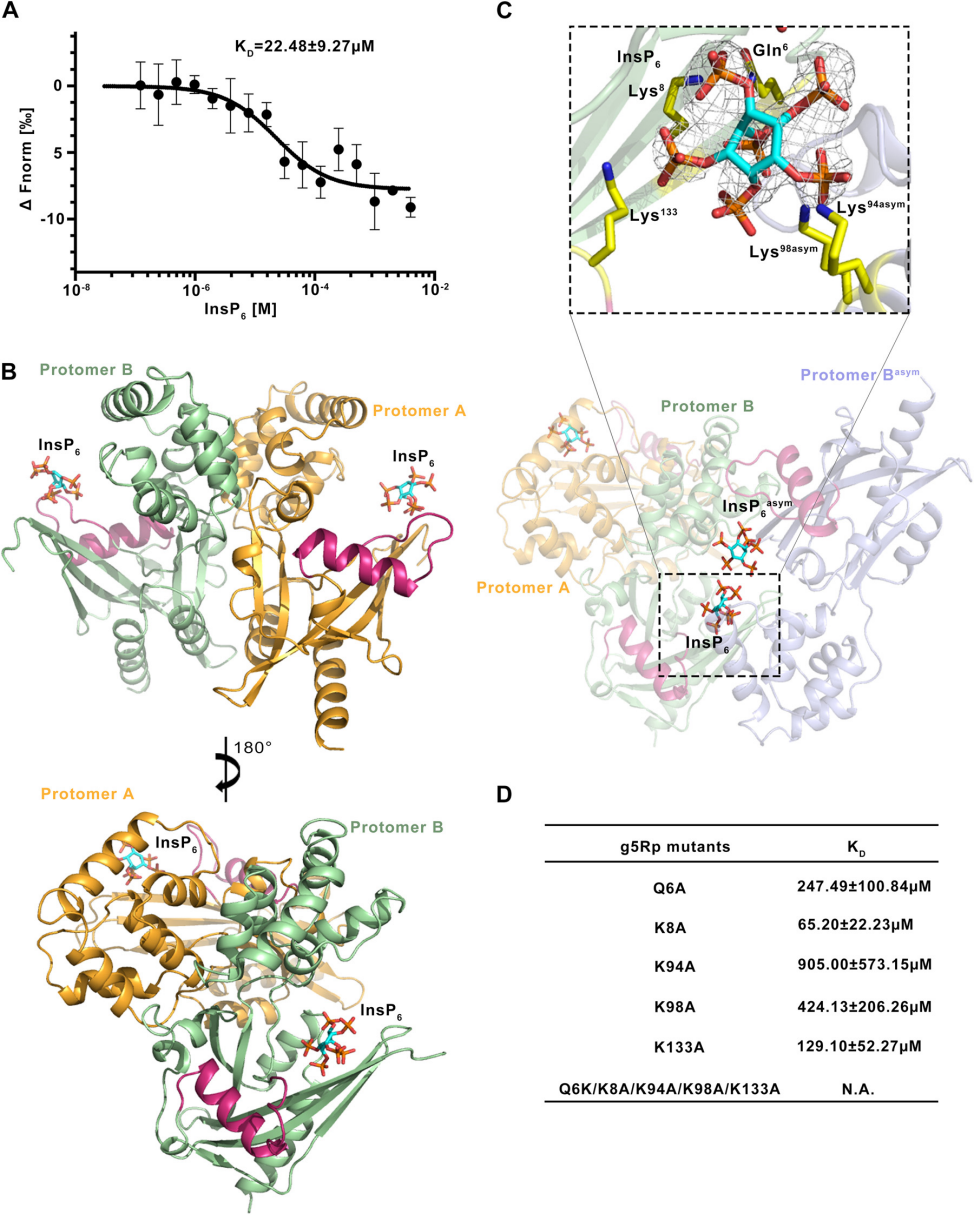
Structure of the g5Rp-InsP_6_ complex. (A) The binding ability of g5Rp with InsP_6_ was measured by MST. The dissociation constant was calculated from three independent replicates (shown as mean ± standard deviation). (B) The dimer of g5Rp in complex with InsP_6_. Protomer A is colored orange, and protomer B is colored pale green; the Nudix motif is colored warm pink. (C) Details of g5Rp in complex with InsP_6_. The electron density of InsP_6_ is shown as the 2Fo-Fc map contoured at 0.8 σ and generated with InsP_6_ omitted. (D) The binding ability between various g5Rp mutants and InsP_6_ was measured by MST. The dissociation constants between g5Rp mutants and InsP_6_ were calculated from three independent replicates (shown as mean ± standard deviation). InsP_6_ is shown as a stick model.

**FIG 7:**
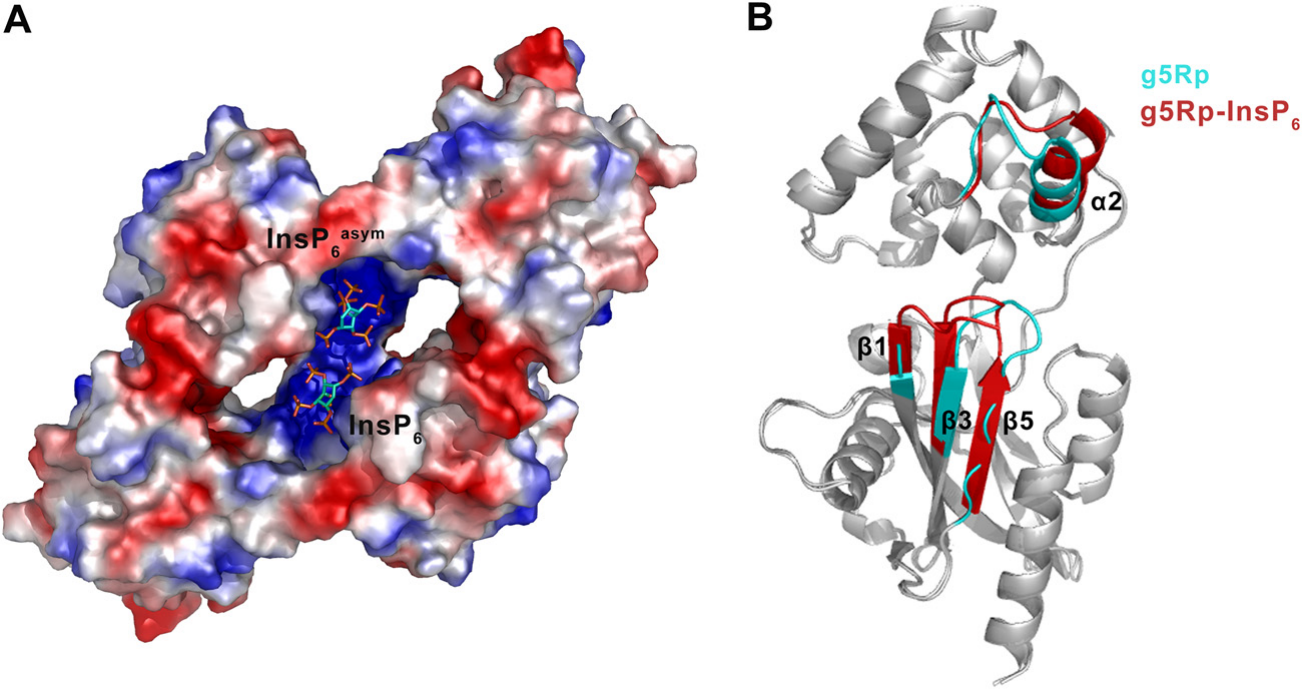
Structural analysis and superposition of the g5Rp-InsP_6_ complex with g5Rp. (A) Surface charge distribution of g5Rp. The range of electrostatic surface potential is shown from −74.712 kT/e in red to +74.712 kT/e in blue. InsP_6_ is shown as a stick model. (B) Superposition of g5Rp with the g5Rp-InsP_6_ complex. The conformational changes are colored in cyan and red, respectively.

g5Rp was originally characterized through its ability to dephosphorylate 5-PP-InsP_5_ (InsP_7_) to produce InsP_6_ (25). We were surprised to find a tight interaction between InsP_6_ and g5Rp by microscale thermophoresis (MST) (Fig. 6A). To gain insight into the molecular basis of the interaction, we determined the crystal structure of the g5Rp-InsP_6_ complex and found that each asymmetric unit contained one g5Rp-InsP_6_ complex in space group *P*4_1_22. PDBePISA analysis revealed that an identical dimeric conformation exists in the g5Rp-InsP_6_ complex structure (Fig. 6B). Two InsP_6_ molecules were situated on the edge of the β1 strand of each g5Rp protomer through interactions with residues Gln^6^, Lys^8^, and Lys^133^ (Fig. 6C). Due to the 2-fold symmetry in the crystal, each of the g5Rp protomers shared two InsP_6_ molecules (InsP_6_ and InsP_6_^asym^) with its neighboring g5Rp protomer in the crystal lattice. Besides the InsP_6_ binding on the β1 strand located on the edge of the Nudix domain, an extra InsP_6_ molecule from the neighboring molecule also interacted with g5Rp through residues Lys^94^ and Lys^98^ on the α5 helix in the helical domain (Fig. 6C). In this way, each InsP_6_ molecule is surrounded by four Lys residues in complex structure. The solvent-accessible surface of the InsP_6_ binding region of g5Rp was calculated according to the electrostatic potential. It was apparent that both InsP_6_ molecules were situated on the highly positively charged area located in the protein cleft between the helical domain and Nudix domain of g5Rp (Fig. 7A). The local conformational changes of g5Rp in the complex structure induced by its interaction with InsP_6_ are illustrated in Fig. 7B. In the complex structure, the β1, β3, and β5 strands located in the Nudix domain had moved closer to the helical domain, and α2 was pushed away from the InsP_6_ binding sites. These changes rendered the g5Rp conformation more stable in the complex.

To assess their relative importance in g5Rp-InsP_6_ interaction, amino acid residues involved in InsP_6_ binding pockets were replaced by single point mutation (Q6A, K8A, K94A, K98A, K133A). Each mutant was tested for its binding affinity for InsP_6_ by MST. Figure 6D showed that mutants resulted in a notable decrease in g5Rp-InsP_6_ interaction, and furthermore, the quintuple mutant Q6A/K8A/K94A/K98A/K133A totally lost the binding ability with InsP_6_. Taken together, the mutagenesis work indicates that positively charged residues Lys8, Lys94, Lys98, and Lys133 form a cluster to mediate the g5Rp-InsP_6_ interaction.

### Analysis of residues involved in g5Rp-RNA interfaces.

**FIG 8:**
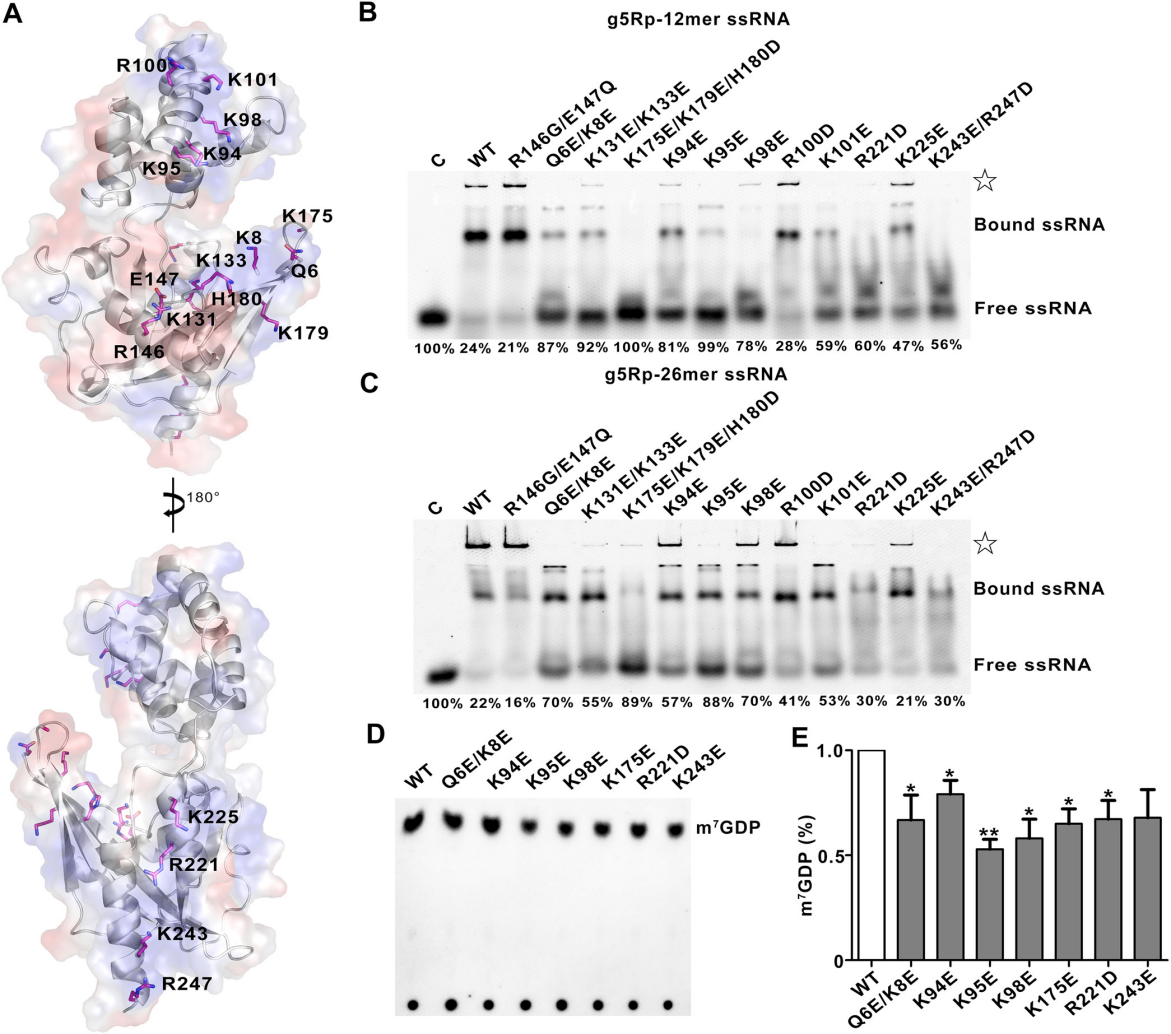
The residues involved in g5Rp-mRNA interfaces and decapping activity. (A) The mutation sites are located in the positively charged regions of g5Rp. (B and C) The binding abilities of g5Rp mutants to 12-mer and 26-mer ssRNAs were determined by EMSA; in the reaction, the protein concentration is 2.00 μM, and the nucleic acid concentration is 0.25 μM; the semiquantitative of the free ssRNA by Image J and the ratio of free ssRNA are marked at the bottom of the picture. The white star represents the precipitation in the gel. (D) The decapping activity of g5Rp mutants. (E) The semiquantitative values of m^7^GDP by Image J and drawing by GraphPad Prism 8 (mean ± standard deviation, *n* ≥ 3; *, *P* < 0.05; **, *P* < 0.01, unpaired *t* test).

To characterize RNA binding surface on g5Rp, we analyzed the electrostatic potential at the surface of g5Rp, which indicated that three highly positively charged areas (areas I to III) may play roles in g5Rp-RNA interaction (Fig. 3A). Area I is located on the helical domain, containing residues Lys94, Lys95, Lys98, Arg100, and Lys101 located on helix α5. Area II is composed of residues Lys8, Lys131, Lys133, Lys135, Arg146, Lys175, Lys179, and His180 mostly located on the β1 and β3 strands, which are close to the Nudix motif; area III is located at the very end of the C terminus of g5Rp, comprising residues Arg^221^, Lys^225^, Arg^226^, Lys^243^, and Lys^247^ on helices α10 and α11 (Fig. 8A). To identify the mRNA binding surfaces on g5Rp further, the residues mentioned above located in three positively charged areas of g5Rp were mutated, respectively. The EMSA pattern showed that some mutants reduce the RNA binding affinity of g5Rp. Specifically, residues Lys^8^, Lys^94^, Lys^95^, Lys^98^, Lys^131^, Lys ^33^, Lys^175^, Arg^221^, and Lys^243^ were critical for single-stranded RNA binding, albeit with different efficiencies (Fig. 8B and C), implying that the g5Rp-RNA interaction interfaces are mainly located at areas I, II, and III. These results also agree with our hypothesis that residues Lys^8^, Lys^94^, Lys^98^, and Lys^133^ of g5Rp are involved in both RNA and InsP_6_ interaction.

We further explored whether these key residues were responsible for cap cleavage in a manner dependent on the RNA moiety interaction. Mutant proteins including Q6E/K8E, K94E, K95E, K98E, K175E, R221D, and K243E were expressed and purified. Consistent with our previous data, incubation of the ^32^P-cap-labeled RNA substrate with wild-type g5Rp resulted in cap cleavage, as observed by m^7^GDP release. When equivalent amounts of the mutants of g5Rp were included in the decapping reaction, the amount of m^7^GDP released was reduced variously in each lane. Mutant K95E decreases the decapping activity almost 50% (Fig. 8D and E), indicating that these residues of g5Rp play a pivotal role in mRNA decapping by interacting with substrate mRNA.

### Residues Gly^132^, Lys^133^, and Glu^147^ in the Nudix motif impact the decapping activity.

**FIG 9:**
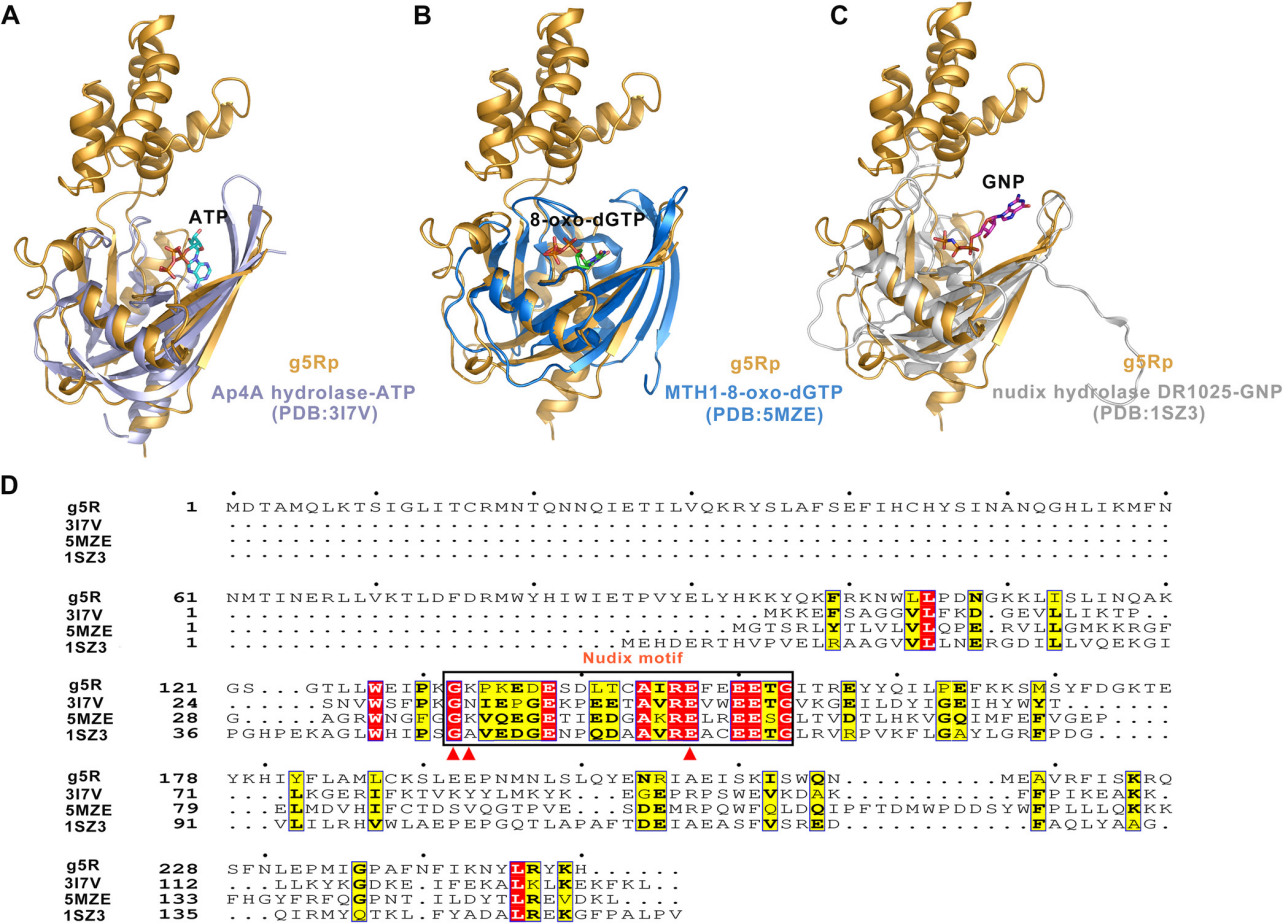
Superposition and sequence alignment of the African swine fever virus decapping enzyme g5Rp with its homologs. (A to C) Superposition of g5Rp with Ap4A hydrolase in complex with ATP (Aquifex aeolicus Vf5, PDB accession no. 3I7V), Nudix hydrolase DR1025 in complex with phosphoaminophosphonic acid-guanylate ester (Deinococcus radiodurans, PDB accession no. 1SZ3), and MTH1 in complex with 8-oxo-dGTP (Mus musculus, PDB accession no. 5MZE). Ap4A hydrolase is shown in slate, Nudix hydrolase DR1025 is shown in marine, and MTH1 is shown in white. ATP, 8-oxo-dGTP, and GNP (GTP substrate analogue, GMPPNP (GNP)) are shown as stick models. (D) Sequence alignment of g5Rp, Ap4A hydrolase, Nudix hydrolase DR1025, and MTH1. The conserved Nudix motif is marked by the black box. The conserved amino acids that bind to the substrates (ATP, 8-oxo-dGTP, and GNP) are marked by red triangles.

The Nudix motif of hydrolases contains crucial residues involved in catalytic activity. However, the residues in the catalytic pocket of g5Rp are still elusive from the viewpoint of structure. To elucidate the function of the key residues in g5Rp, three substrate binding structures from the Nudix superfamily were selected to identify homologous domains with high similarity at the potential catalytic pockets (Fig. 9A to C), as shown in Table 2, *viz.*, Ap4A hydrolase (Aquifer aeolicus, PDB accession no. 3I7V) (33), Nudix hydrolase DR1025 (Deinococcus radiodurans, PDB accession no. 1SZ3), and MTH1 (Mus musculus, PDB accession no. 5MZE) (34, 35) (all belonging to the Nudix superfamily). Superposition of the C terminus of g5Rp with that of MTH1, Ap4A hydrolase, and Nudix hydrolase DR1025 resulted in Cα backbone root mean square deviation values of 0.50, 3.08, and 5.6 Å, respectively, despite the low sequence identities among these proteins (Fig. 9D). Therefore, the potential substrate binding site of g5Rp was proposed on the basis of the superpositions of these substrate binding protein structures of the Nudix superfamily. Residues Gly^132^, Lys^133^, and Glu^147^ located on the Nudix motif of g5Rp may be responsible for cap cleavage.

**TABLE 2:** Data collection and refinement statistics

Parameter or statistic	g5Rp	g5Rp-InsP_6_
Data collection		
Resolution (Å)	2.50	2.25
Space group	*P*2_1_2_1_2	*P*4_1_22
Unit-cell parameters (Å, °)	*a *= 56.1, *b *= 105.1, *c *= 49.5, α = β = γ = 90	*a *= 48.4, *b *= 48.4, *c *= 220.1 α = β = γ = 90
Resolution (Å)^*a*^	50.00–2.5 (2.54–2.5)	50.00–2.25 (2.29–2.25)
* R*_merge_^*b*^ (%)	12.7 (30.5）	6.7 (53.0）
* R_pim_*^*c*^ (%)	4.1 (15.7)	1.7 (14.7)
Avg *I/*σ (*I*)	20 (2.1)	77.5 (12.4)
Multiplicity	9.1 (3.4)	15 (12.2)
No. of observed reflections	9,721 (248)	13,490 (668)
No. of unique reflections	9,678 (506)	13,350 (1,285)
Completeness (%)	91.7 (48.80)	99.37 (98.39)
Matthews coefficient (Å^3^ Da^−1^)	2.65	2.35
Solvent content (%)	53.68	47.61
CC_1/2_	0.97 (0.92)	1.00 (0.98)
Molecules per asymmetric unit	1	1

Refinement		
Resolution (Å)	50.00–2.50	50.00–2.25
* R*_work_/*R*_free_	0.2037/0.2744	0.1724/0.2307

MolProbity		
Ramachandran favored (%)	92.59	96.73
Ramachandran allowed (%)	7.00	3.27
Ramachandran outliers (%)	0.41	0.00
Rotamer outliers (%)	2.20	0.00
Clashscore	23.08	5.02
Overall score	6.1	4.5

Avg B factor (Å^2^)	64.27	48.25
Protein	64.32	46.61
Water	44.94	46.12
Ligand	0	148.4

No. of atoms		
Protein	2,026	2,111
Water	28	106
Wilson B value	43.01	37.45

RMSDs^*d*^		
Bond lengths (Å)	0.010	0.008
Bond angles (°)	1.186	0.925

a Numbers in the parentheses are for the highest-resolution shell.

b $R_{\text{merge}}=\sum_{\text{hkl}}\sum_{i}\left|I_{i}(\text{hkl})–〈I(\text{hkl})〉\right|/\sum_{\text{hkl}}\sum_{i}I_{i}(\text{hkl})$, where *I_i_*(*hkl*) is an individual intensity measurement and *I*(*hkl*) is the average intensity for all *i* reflections.

c *R*_pim_ is approximated by multiplying the *R*_merge_ value by the factor [1/(*N* – 1)]^1/2^, where *N* is the overall redundancy of the data set.

d RMSDs, root mean square deviations.

**FIG 10:**
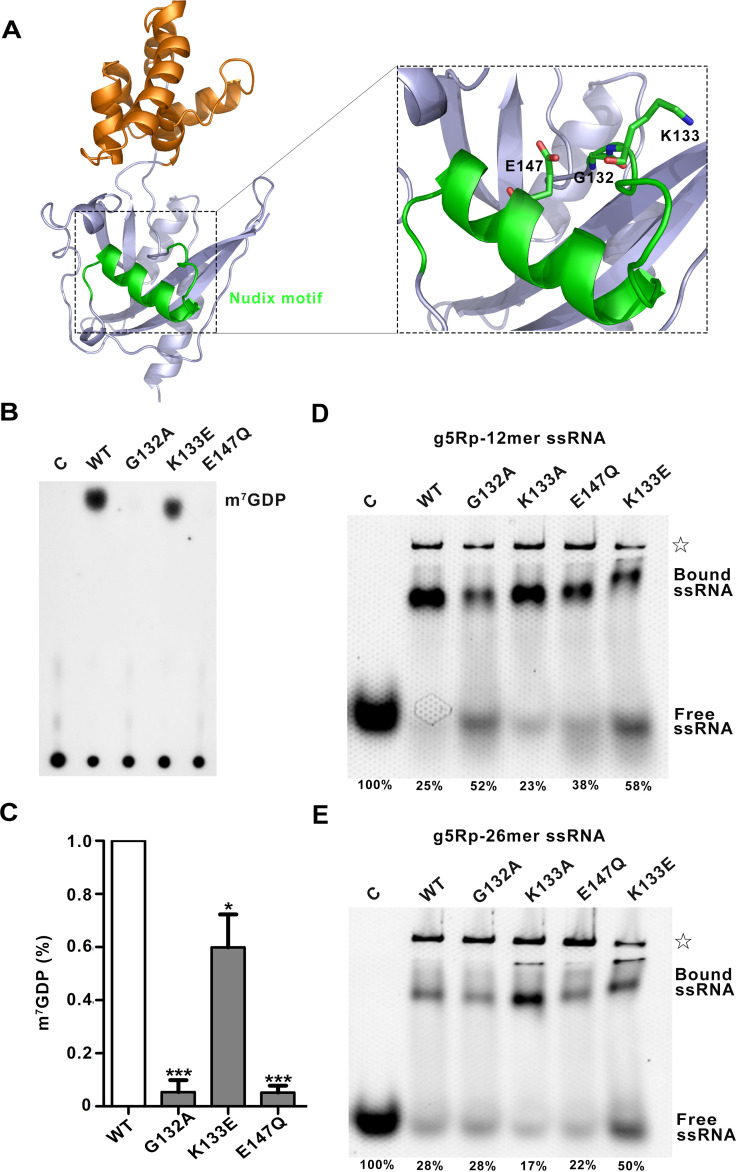
The Nudix motif of g5Rp involved in mRNA binding and decapping. (A) The details of the Nudix motif in g5Rp. The Nudix motif is colored green. The mutant residues are shown as sticks. (B) The decapping activity of g5Rp mutants. (C) The semiquantitative of m^7^GDP by Image J and drawing by GraphPad Prism 8 (mean ± standard deviation, *n* ≥ 3; *, *P* < 0.05; ***, *P* < 0.001, unpaired *t* test). (D and E) The binding abilities of g5Rp mutants to 12-mer and 26-mer ssRNAs were determined by EMSA; in this reaction, the protein experimental concentration is 2.00 μM, and the nucleic acid experimental concentration is 0.25 μM. The semiquantitative of the free ssRNA by Image J and the ratio of free ssRNA are marked at the bottom of the picture. The white star represents the precipitation in the gel.

To investigate the potential roles of these key residues located on the Nudix motif in the decapping activity, we replaced g5Rp residues G132, K133, and E147 from the Nudix motif (Fig. 9D and Fig. 10A) with Ala, Glu, and Gln, respectively. As expected, the replacement of the residue K133 with glutamate resulted in a 30% decrease in the decapping activity. And the replacement of the residues G132 and E147 by alanine and glutamine, respectively, inactivated the decapping function of g5Rp completely (Fig. 10B and C). No m^7^GDP was observed when the two mutants of g5Rp were included in the decapping reaction, validating that the decapping activity was dependent on these two key residues located in the Nudix hydrolase motif. Interestingly, EMSA results showed that mutant K133E reduces g5Rp’s binding affinity to RNA, which suggests that the loop region of the Nudix motif takes part in substrate mRNA binding (Fig. 10D and E).

### InsP6 inhibits the decapping activity by disrupting g5Rp-mRNA interaction.

**FIG 11:**
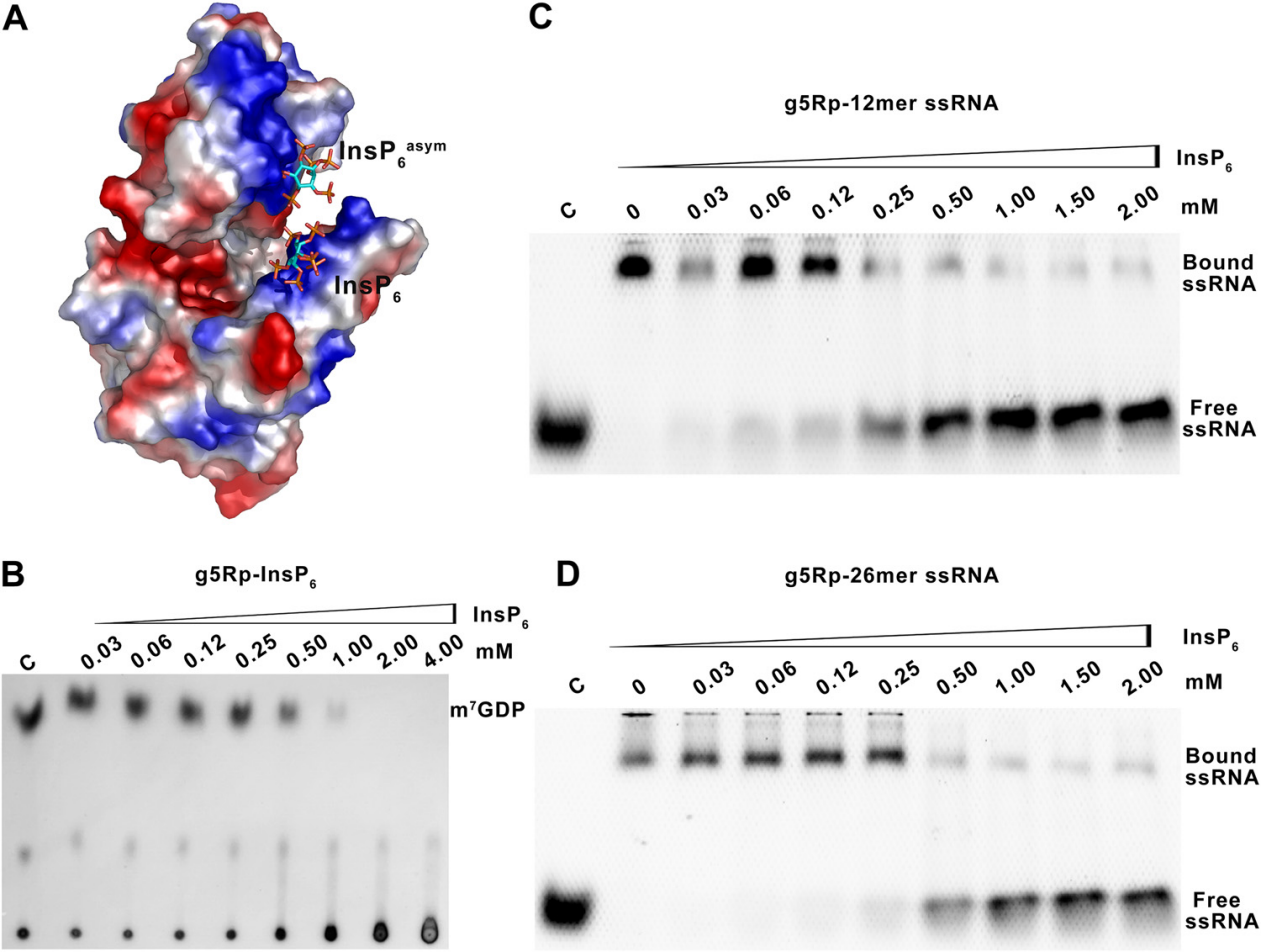
The influence of InsP_6_ on mRNA binding and decapping activity of g5Rp. (A) The surface charge of g5Rp in complex with InsP_6_. (B) The influence of InsP_6_ on decapping activity of g5Rp. (C and D) The influence of InsP_6_ on binding abilities of g5Rp with 12-mer and 26-mer ssRNAs was determined by EMSA, respectively. The different concentrations of InsP_6_ with g5Rp in the reaction system are indicated above the gel; the concentrations of protein and nucleic acid were 2.00 μM and 0.25 μM, respectively.

The finding that residues located on mRNA binding regions of g5Rp are also playing pivotal roles in g5Rp-InsP_6_ interaction suggests that InsP_6_ may inhibit the g5Rp decapping activity through preventing g5Rp from binding to its mRNA substrate (Fig. 11A). This prediction was confirmed by decapping and EMSAs using recombinant g5Rp, InsP_6_, and RNA substrates *in vitro*. Increasing amounts of InsP_6_ were added to the decapping reactions to analyze its effect on RNA decapping by g5Rp. As shown in Fig. 11B, the addition of InsP_6_ significantly affected g5Rp cleavage, suggesting that InsP_6_ can inhibit the decapping activity of g5Rp *in vitro*. To investigate if this inhibitory mechanism of InsP_6_ on g5Rp is due to inositol phosphate competitively inhibiting mRNA binding to the g5Rp, we further measured the competition of InsP_6_ with nucleic acids for the binding to g5Rp by using EMSA. As expected, the amount of free single-stranded nucleic acids increased with an increasing concentration of InsP_6_, demonstrating that InsP_6_ interrupts the g5Rp-mRNA interaction through directly binding to g5Rp (Fig. 11C and D). In addition, all residues involved in InsP_6_ interaction in g5Rp were mutated into alanine at the same time. The quintuple mutant (Q6A/K8A/K94A/K98A/K133A) of g5Rp lost most of its ability to bind with both InsP_6_ and RNA (Fig. 6D and see Fig. 13A). These mutations also significantly affected the catalytic ability of g5Rp *in vitro* (see Fig. 13C and D), suggesting that InsP_6_ inhibits the mRNA-decapping activity of g5Rp through competing for the substrate mRNA binding surface in g5Rp.

### Transient expression of g5Rp decreases levels of mRNA substrates in 293T cells.

**FIG 12:**
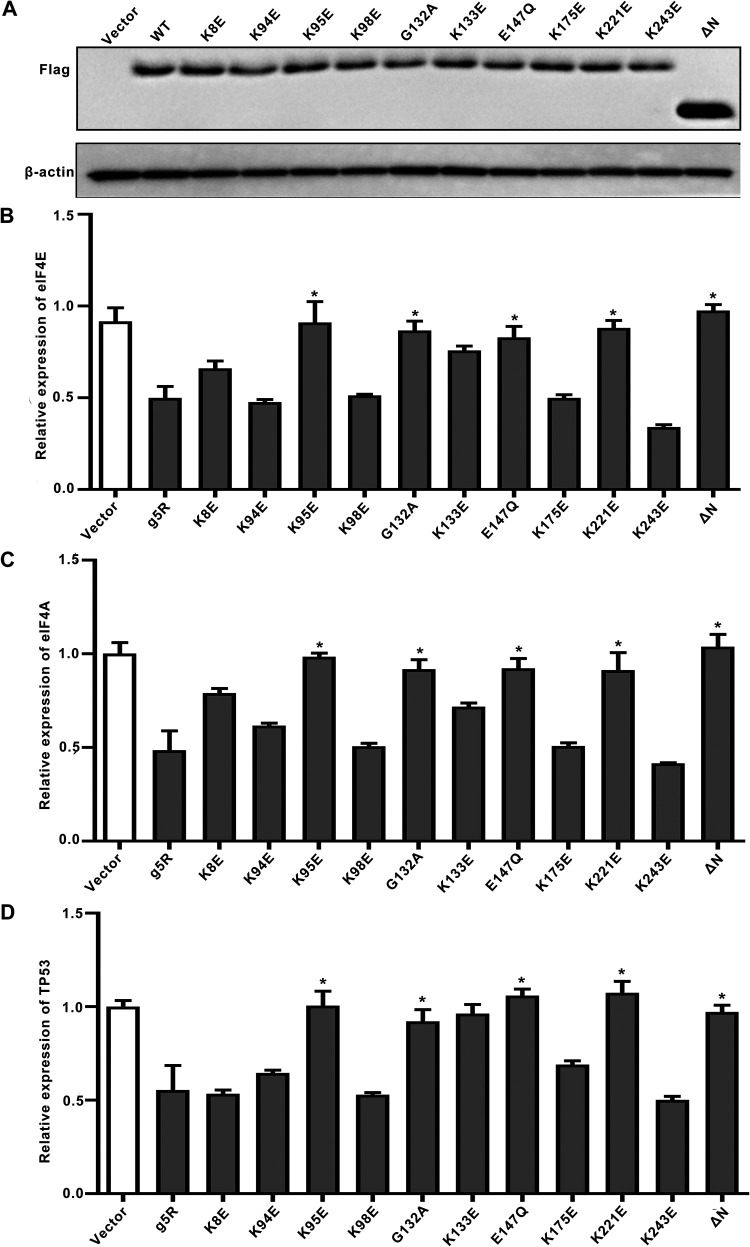
The influence of g5Rp and mutants on mRNA substrate level in 293T cells. (A) The expression of various g5Rp mutants in 293T cells was analyzed by Western blotting. (B) The mRNA levels of eIF4E in 293T cells when a series of g5Rp mutants was overexpressed. (C) The mRNA level of eIF4A in 293T cells when a series of g5Rp mutants was overexpressed. (D) The mRNA level of TP53 in 293T cells when a series of g5Rp mutants was overexpressed (mean ± standard deviation, *n* ≥ 3; *, *P* < 0.05; **, *P* < 0.01, unpaired *t* test).

The above data provide strong *in vitro* evidence for g5Rp-mRNA interaction being a critical step for the decapping enzyme process. To determine whether changes in g5Rp-mRNA interaction were directly related to the stability of cellular mRNAs *in vivo*, representative cellular mRNA (eIF4E, eIF4EA, and TP53) levels were tested by quantitative real-time PCR (RT-qPCR) in cells. In 293T cells, the Flag-tagged g5Rp and the g5Rp mutants (K8E, K94E, K95E, K98E, G132A, K133E, E147Q, K175E, R221D, and K243E) were overexpressed, respectively. As shown in Fig. 12A, the g5Rp-WT and mutant proteins were detected by Western blotting. The mRNA levels of target genes (eIF4E, eIF4EA, and TP53) were decreased in 293T cells overexpressing g5Rp-WT. There were no obvious changes in mRNA levels in the catalytic destructive mutants Q132A and E147Q. The overexpression in cells of truncated version g5Rp-ΔN and mutants K95E and R221D, mutants which significantly lost the RNA binding ability *in vitro*, had no effect on the mRNA levels of target genes in 293T cells. Mutants K8E and K133E, which had reduced RNA binding *in vitro*, had various degrees of increase compared with the mRNA levels of the g5Rp-WT group. However, the changes in mRNA levels of target genes observed in mutants K94E, K98E, K175E, and K243E did not have statistical differences from those in g5Rp-WT (Fig. 12B to D). Taken together, these results suggest that key residues K8, K95, K133, and R221, playing pivotal roles in g5Rp-RNA interaction, are also important to the g5Rp-related cellular RNA degradation *in vivo*.

## DISCUSSION

Given that an ASFV outbreak in China would potentially devastate the world’s largest pork producer, significant efforts have been made to determine the structures and functions of essential viral proteins that may be used as targets for new anti-ASFV drugs. Several structures of ASFV-encoded enzymes and associated proteins that are involved in viral transcription and replication have been reported, including AP endonuclease (36), the histone-like protein pA104R (37), pS273R protease (38), DNA ligase (39), and dUTPase (40, 41). However, the structures and functions of some critical ASFV proteins remain elusive, including those of g5Rp, a decapping enzyme that is crucial for viral infection (23). Our structures of g5Rp alone and in complex with InsP_6_ provide the molecular basis for g5Rp substrate recognition and reveal that inositol phosphate was involved in the regulation of cellular mRNA degradation through direct interaction with the ASFV decapping enzyme g5Rp. Three potential RNA binding regions are identified, including a novel folding domain located on the helical domain of g5Rp and the Nudix motif on its C terminus. More importantly, identification of the major nucleic acid binding surfaces as well as the binding pocket of InsP_6_ on g5Rp provides important structural information and a novel strategy for future anti-ASFV drug design.

**FIG 13:**
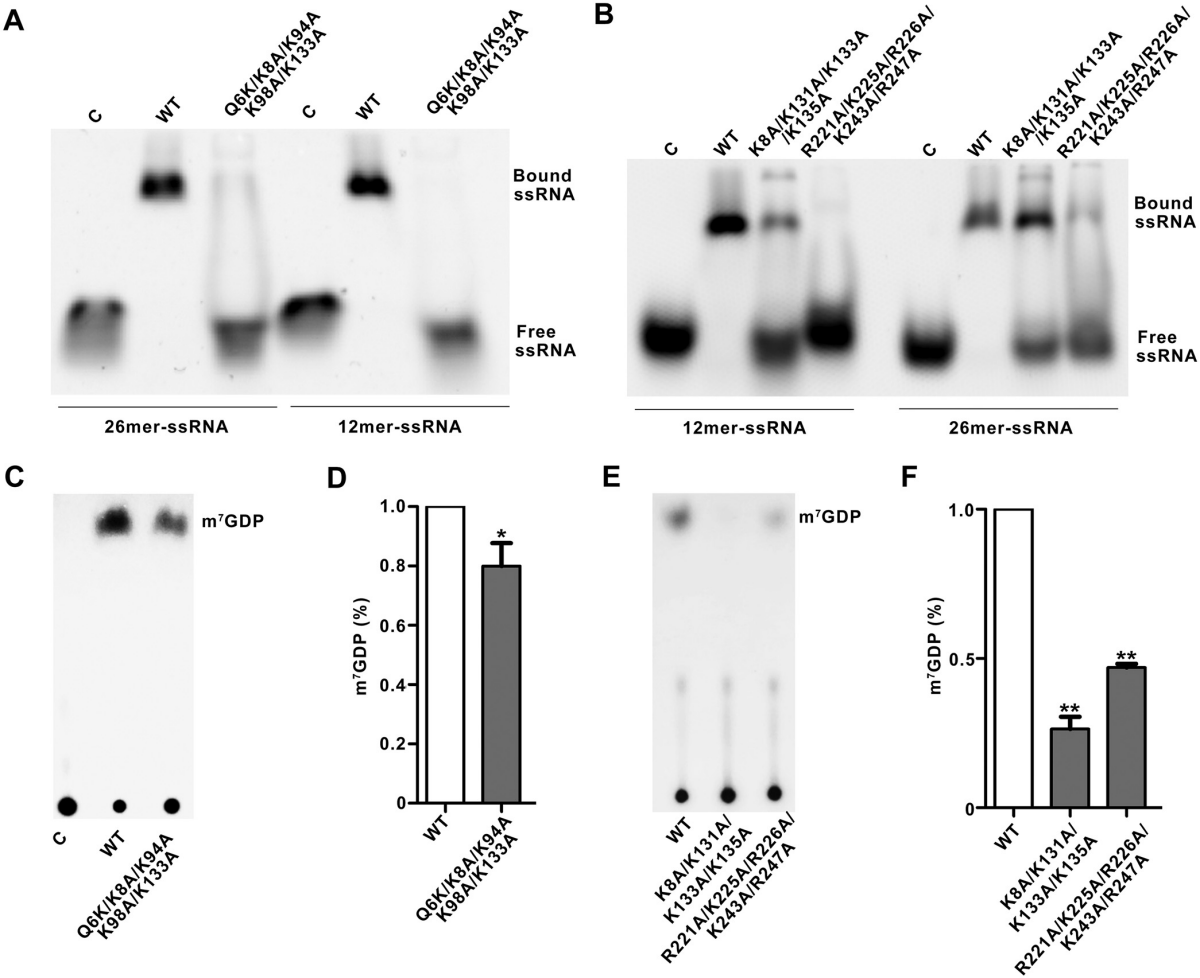
The decapping activity and RNA binding abilities of g5Rp mutants. (A) The comparison in binding abilities of g5Rp and Q6K/K8A/K94A/K98A/K133A (final concentration, 2.00 μM) with 26-mer and 12-mer ssRNAs (final concentration, 0.25 μM). (B) The binding abilities of K8A/K131A/K133A/K135A and R221A/K225A/R226A/K243A/R247A (final concentration, 2.00 μM) with 12-mer and 26-mer ssRNAs (final concentration, 0.25 μM). (C and E) The decapping activities of g5Rp mutants (Q6K/K8A/K94A/K98A/K133A, K8A/K131A/K133A/K135A, and R221A/K225A/R226A/K243A/R247A). (D and F) The semiquantitative of m^7^GDP by GraphPad Prism 8 (mean ± standard deviation, *n* ≥ 3; *, *P* < 0.05; **, *P* < 0.01, unpaired *t* test).

To explore the nucleic acid binding properties of g5Rp, we conducted a series of nucleic acid binding experiments. Results indicated that an intact dimeric interface is efficient for g5Rp-RNA interaction. Meanwhile, the helical domain and Nudix domain of g5Rp are both involved in ssRNA interaction. Our EMSA and SPR measurements show that the helical domain of g5Rp can bind with ssRNA with equally high affinity as the full-length protein. Six α-helices form a globin-fold-like helical domain, which is different from the traditional RNA binding domain that prefers to adopt the alpha/beta topologies (42–45). According to the g5Rp structure, the surface electrostatic potential characteristics of the N terminus present a highly positively charged area on helix α5. The single point mutations of positively charged residues in the N terminus significantly reduced the nucleic acid binding activity of g5Rp with ssRNA (Fig. 8B and C). Furthermore, there are two positive areas located on the C terminus of g5Rp, including the Nudix motif, participating in the substrate RNA interaction. We mutated the two positively charged regions (K8A/K131A/K133A/K135A and R221A/K225A/R226A/K243A/R247A) located in the Nudix domain; the EMSA data showed that the nucleic acid binding ability of these two mutants was significantly reduced (Fig. 13B), and the Fig. 13E and F data showed that the substantial decline in capacity of K8A/K131A/K133A/K135A removed the m^7^Gppp RNA cap. These results predicated that the Nudix motif of g5Rp possesses substrate selectivity at the step of mRNA binding.

Previously, studies revealed that the Nudix motif (residues 132 to 154) is an essential component of the α-β-α sandwich in the catalytic center of g5Rp. Several of the conserved catalytic amino acids and glutamate residues (E^147^, E^149^, E^150^, and E^151^) located on the α-helix of the Nudix motif of g5Rp have been found to be important for the activity of Nudix hydrolases (23, 28). However, the function of the loop region within the Nudix motif is exclusive, leading us to predicate that the loop region may contribute to binding with mRNA. Therefore, we mutated several residues in this loop region, including the mutations K133E and G132A, and examined the effects on the protein’s interaction with single-stranded nucleic acids. It is interesting to find that substitutes for the conserved residues K^133^ and G^132^ are highly sensitive to g5Rp-RNA interaction. Compared with glutamate residues located on the α-helix of the Nudix motif of g5Rp involved in mRNA cap structure interaction, residues K^133^ and G^132^ are important for binding with the RNA moiety on the substrate. In this way, we provided a demonstration that the short loop in the Nudix motif is required for g5Rp-RNA interaction. Including the Nudix motif, three positively charged patches on the g5Rp surface were mapped as mRNA binding regions. Furthermore, we also investigated the importance of the residues involved in mRNA interaction in g5Rp-mediated decapping. The g5Rp mutants K8E, K94E, K95E, K98E, K175E, and R221D showed a strong reduction in decapping activity, demonstrating the importance of the mRNA binding residues for catalysis. The dimeric form of g5Rp is also important to the decapping activity. We constructed mutant g5Rp-I84A/I116A/L200A/I206A/F222A in which the dimerization surface was destroyed. mRNA-decapping assays showed that the decapping activity of mutant g5Rp-I84A/I116A/L200A/I206A/F222A decreased drastically (Fig. 5F). It will be of profound interest to elucidate the structural basis of the enzymatic activity of g5Rp by solving the structure of g5Rp in complex with mRNA in the future.

**FIG 14:**
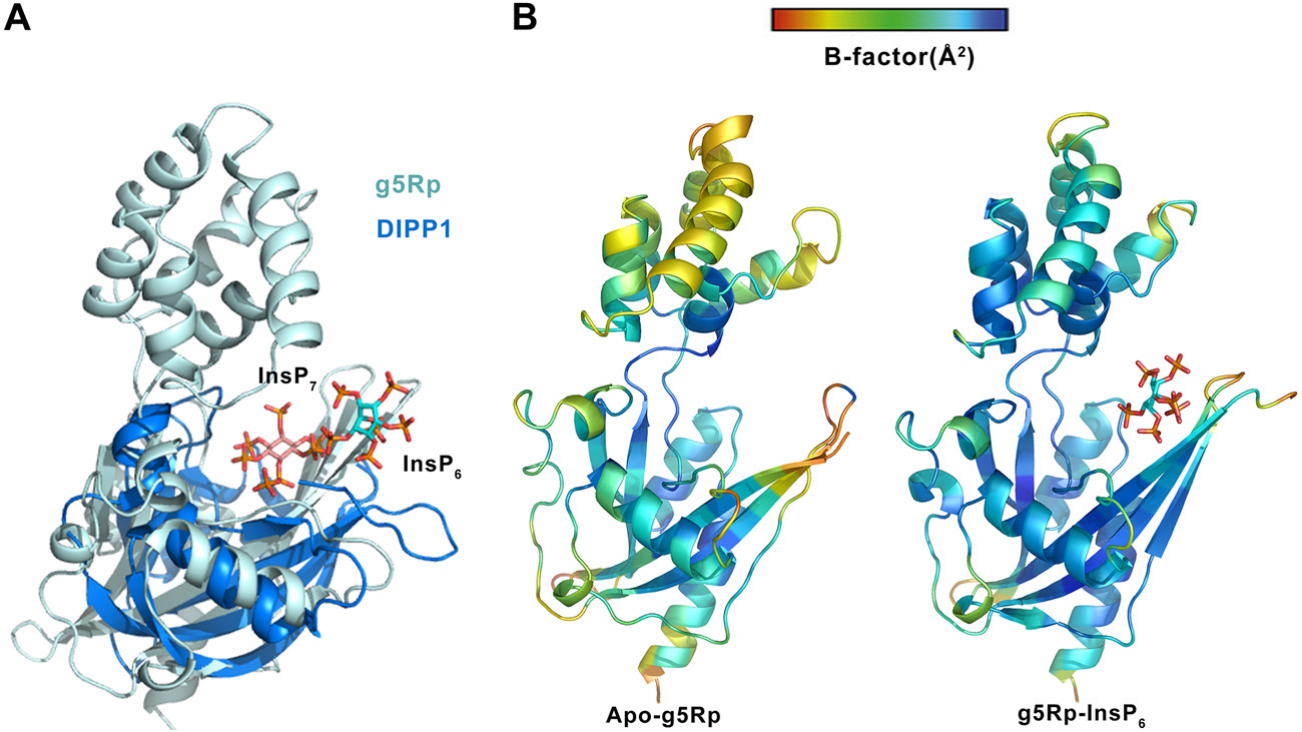
Superposition of g5Rp-InsP_6_ with DIPP1-InsP_7_ and the thermal parameter (B factor) distribution in g5Rp and g5Rp-InsP_6_ complex. (A) Superposition of g5Rp-InsP_6_ with DIPP1-InsP_7_ (Homo sapiens, PDB accession no. 6PCL). g5Rp is shown in pale cyan, and MTH1 is shown in marine. InsP_6_ and InsP_7_ are shown as stick models. (B) B factor distribution in apo-g5Rp and g5Rp-InsP_6_, shown as implemented by PyMOL. The Cα B factors are depicted on the structure in dark blue (lowest B factor) through to red (highest B factor), with the radius of the ribbon increasing from low to high B factor. The lower B factor is observed in the overall structure of g5Rp-InsP_6_, with the InsP_6_ binding sites also displaying lower-than-average B factors, consistent with the InsP_6_ contacts stabilizing this region of g5Rp relative to the overall structure.

The other important finding in this study was that InsP_6_ is able to inhibit the decapping activity of g5Rp. As we know, InsP_6_ is widespread in cells with diverse biological functions (46–49). Here, we found that InsP_6_ competes with mRNA substrates for binding to g5Rp and inhibits its decapping activity. A previous study reported that g5Rp is a diphosphoinositol polyphosphate phosphohydrolase (DIPP), which preferentially removes the 5-β-phosphate from InsP_7_ to produce InsP_6_ with unclear functional significance (25). Later, Parrish and colleagues identified that g5Rp can hydrolyze the mRNA cap when tethered to an RNA moiety *in vitro* (23). Our results show that InsP_6_ as the product of g5Rp playing the role of DIPP can directly inhibit the mRNA-decapping activity of g5Rp. To illustrate the structural basis of the inhibitory mechanism of InsP_6_ for the decapping activity of g5Rp, we solved the structure of the complex of g5Rp with InsP_6_ and also the enzyme-product complex in the Nudix superfamily. To our surprise, InsP_6_ is located on the mRNA binding region instead of in the catalytic center of the g5Rp. Furthermore, we superposed the catalytic domain of g5Rp-InsP_6_ complex with the structures of human DIPP1 in complex with the substrate InsP_7_ (50, 51). The visualized result showed that the substrate InsP_7_ is located in the catalytic center of DIPP1, unlike InsP_6_, which sits on the edge of the catalytic domain of g5Rp (Fig. 14A). Therefore, the structure of the g5Rp-InsP_6_ complex may represent an intermediate in the release of the product of the enzymatic reaction (52). We also noticed that InsP_6_ decreased the temperature value (B factor) around the binding sites compared with B factor in the same regions of the g5Rp wild-type structure, suggesting that the flexible loop closed to the catalytic center is locked in place by InsP_6_ (Fig. 14B). InsP_6_ itself was refined with a correspondingly high B factor that exceeded the average B factor of the protein in complex. Considering that the g5Rp-InsP_6_ interaction has a dissociation constant (*K_d_*) in the 22.5 μM range, the ligand achieves only a reasonable occupancy of 70% (53). To avoid an instance of overenthusiastic interpretation of ligand density, we tested the InsP_6_ binding site by using single point mutations. Residues involved in the InsP_6_ binding surface of g5Rp replaced by alanine (Q6A/K8A/K94A/K98A/K133A) reduced its InsP_6_ binding capacity and RNA interaction, indicating the destructive InsP_6_ binding site has the capability to abolish the substrate RNA binding ability of g5Rp (Fig. 13A, C, and D).

Our study raises the possibility that g5Rp hydrolyzes InsP_7_ to upregulate the level of InsP_6_, which is a key regulator of g5Rp-mediated mRNA decapping during ASFV infection *in vivo* (54). Very recently, Sahu and colleagues reported that InsP_7_ regulates the NUDT3-mediated mRNA decapping and also observed the phenomenon that InsP_6_ inhibits mRNA decapping by NUDT3 (54). There are emerging signs that the functions of InsP_6_ are associated with mRNA transportation and degradation in ASFV-infected cells. Further studies on the function of InsP_6_ and the regulation mechanism in the inositol-based cell signaling family during viral infection are required.

## MATERIALS AND METHODS

### Cell culture.

The human 293T cells were cultured in Dulbecco’s modified Eagle’s medium (DMEM) (HyClone) supplemented with 10% fetal bovine serum (FBS) (Gibco), 100 U/mL penicillin, and 100 μg/mL streptomycin at 37°C under a humidified 5% CO_2_ atmosphere (Thermo).

### Plasmid construction, protein expression, and purification.

The gene encoding ASFV g5Rp (*D250R*) was synthesized and subcloned into pSMART-1 and pcDNA3.1, respectively. The amino acid sequence of g5Rp comes from UniProt (https://www.uniprot.org/), with accession no. P32092. The point mutants and truncation variants of g5Rp (*viz.*, Q6E/K8E, K94E, K95E, K98E, R100D, K101E, K131E/K133E, R146G/E147Q, K175E/K179E/H180D, R221D, K225E, K243E/R247D, Q6A/K8A/K94A/K98A/K133A, K8A/K131A/K133A/K135A, R221A/K225A/R226A/K243A/R247A, I84A/I116A/L200A/I206A/F222A, g5Rp-ΔC [helical domain, residues 36 to 124], and g5Rp-ΔN [Nudix domain, connecting residues 1 to 35 and 125 to 250 directly]) were generated using the Fast Mutagenesis V2 kit (Vazyme Biotech, China). The primers used in this study are listed in Table 3. The recombinant plasmids were confirmed by sequencing (Sangon Biotech, China) before being introduced into E. coli BL21(DE3) (Invitrogen, USA) or human 293T cells. The bacterial cells were cultured in Luria broth medium at 35°C until the optical density at 600 nm reached 0.6 to 0.8. Protein expression was then induced by the addition of isopropyl-β-d-1-thiogalactopyranoside for 16 h at 16°C. The g5Rp molecules were purified by Ni-nitrilotriacetic acid (NTA) (Qiagen, Germany) affinity chromatography, followed by heparin affinity chromatography (GE Healthcare, USA). The peak fractions containing the target proteins were pooled, concentrated to 1 mL, and finally loaded onto a Superdex 75 column (GE Healthcare, USA) for further purification and characterization. Selenomethionine-labeled g5Rp (SeMet-g5Rp) was then prepared using a previously described protocol (55). The purity of all proteins was above 95% on the SDS-PAGE gel.

**TABLE 3:** Primer sequences for generation of the g5Rp mutants

Primer name	Sense sequence (5′–3′)	Orientation
R146G/E147Q-F	ATCTGACCTGTGCAATTGGCCAATTTGA	Forward
R146G/E147Q-R	GGCCAATTGCACAGGTCAGATCGCTTTC	Reverse
Q6E/K8E-F	GAGCTGGAAACCAGTATTGGTCTGATTACCTGCCGT	Forward
Q6E/K8E-R	CCAATACTGGTTTCCAGCTCCATGGCGGTATCCAT	Reverse
K131E/K133E-F	GAAATTCCGGAAGGTGAACCGAAAGAAGATGAAA	Forward
K131E/K133E-R	CACCTTCCGGAATTTCCCACAGCAGGGTGCCACT	Reverse
K175E/K179E/H180E-F	GTGAAACCGAATATGAAGACATCTATTTCCTGGCCA	Forward
K175E/K179E/H180E-R	GTCTTCATATTCGGTTTCACCGTCAAAATAGCTCAT	Reverse
K94E-F	GTTTATGAACTGTATCATGAAAAATACCAGAAGT	Forward
K94E-R	CATGATACAGTTCATAAACCGGGGTTTCAATCC	Reverse
K95E-F	TATGAACTGTATCATAAAGAATACCAGAAGTTCC	Forward
K95E-R	CTTTATGATACAGTTCATAAACCGGGGTTTCAA	Reverse
K98E-F	TATCATAAAAAATACCAGGAGTTCCGCAAAAATT	Forward
K98E-R	CCTGGTATTTTTTATGATACAGTTCATAAACCGG	Reverse
R100D-F	AAAAAATACCAGAAGTTCGACAAAAATTGGCTGCT	Forward
R100D-R	TCGAACTTCTGGTATTTTTTATGATACAGTTCATA	Reverse
K101E-F	AAATACCAGAAGTTCCGCGAAAATTGGCTGCTGC	Forward
K101E-R	CGCGGAACTTCTGGTATTTTTTATGATACAGTTC	Reverse
R221D-F	CAGAATATGGAAGCCGTTGATTTTATTAGCAAACG	Forward
R221D-R	TCAACGGCTTCCATATTCTGCCAACTAATTTTGCT	Reverse
K225E-F	GCCGTTCGTTTTATTAGCGAACGTCAGAGCTTTA	Forward
K225E-R	CGCTAATAAAACGAACGGCTTCCATATTCTGCCA	Reverse
K243E/R247D-F	TATTGAGAATTATCTGGACTACAAGCATTAA	Forward
K243E/R247D-R	TCCAGATAATTCTCAATAAAATTGAATGCCGGACCA	Reverse
g5Rp-ΔN-F	GTGCAGAAACGTTATAGTCTGCTGTGGGAAATTCCG	Forward
g5Rp-ΔN-R	CGGAATTTCCCACAGCAGACTATAACGTTTCTGCAC	Reverse
g5Rp-ΔC-F	CTGGTGCCGCGCGGCAGCCTGGCATTTTCAGAATTC	Forward
g5Rp-ΔC-R	GATGATGATTCCTCCTCCGGTGCCACTACCTTTTGC	Reverse
G132A-F	CCGAAAGCTAAACCGAAAGAAGATGAAAGCGATCTG	Forward
G132A-R	TTTCGGTTTAGCTTTCGGAATTTCCCACAGCAGGGT	Reverse
K133A-F	GAAAGGTGCACCGAAAGAAGATGAAAGCGATCTGAC	Forward
K133A-R	TCTTTCGGTGCACCTTTCGGAATTTCCCACAGCAGG	Reverse
Q6A-F	GCGCTGAAAACCAGTATTGGTCTGATTACCTGC	Forward
Q6A-R	AATACTGGTTTTCAGCGCCATGGCGGTATCCAT	Reverse
K8A-F	CTGGCAACCAGTATTGGTCTGATTACCTGCCGTATG	Forward
K8A-R	ACCAATACTGGTTGCCAGCTGCATGGCGGTATCCAT	Reverse
K94A-F	TTATGAACTGTATCATGCAAAATACCAGAAGTTCCG	Forward
K94A-R	GCATGATACAGTTCATAAACCGGGGTTTCAATCCAA	Reverse
K131A/K133A/K135A-F	GCAGGTGCACCGGCAGAAGATGAAAGCGATCTGACC	Forward
K131A/K133A/K135A-R	TTCTGCCGGTGCACCTGCCGGAATTTCCCACAGCAG	Reverse
R221A/K225A/R226A-F	TGCTTTTATTAGCGCAGCTCAGAGCTTTAATCTGGA	Forward
R221A/K225A/R226A-R	GCTGCGCTAATAAAAGCAACGGCTTCCATATTCTGC	Reverse
K243A/R247A-F	TATTGCGAATTATCTGGCCTACAAGCATTAA	Forward
K243A/R247A-R	GCCAGATAATTCGCAATAAAATTGAATGCCGGACCA	Reverse
I84A-F	TATTTGGGCTGAAACCCCGGTTTATGAACTGTAT	Forward
I84A-R	GGGGTTTCAGCCCAAATATGATACCACATGCGAT	Reverse
I116A-F	TAGTCTGGCCAACCAGGCAAAAGGTAGTGGCA	Forward
I116A-R	GCCTGGTTGGCCAGACTAATCAGTTTTTTACCAT	Reverse
L200A-F	ATATGAATCTGAGTGCGCAGTATGAAAATCGC	Forward
L200A-R	GCGCACTCAGATTCATATTCGGTTCTTCCAGGCT	Reverse
I206A-F	GCTGCCGAAATTAGCAAAATTAGTTGGCAGAATA	Forward
I206A-R	TTTGCTAATTTCGGCAGCGCGATTTTCATACTGC	Reverse
F222A-F	CGTTCGTGCTATTAGCAAACGTCAGAGCTTTA	Forward
F222A-R	TTGCTAATAGCACGAACGGCTTCCATATTCTGCC	Reverse

### Protein crystallization and optimization.

The prepared SeMet-g5Rp was concentrated to 12 mg/mL for the crystallization trials. The crystals were grown using the hanging-drop vapor diffusion method at 16°C in a reservoir solution containing 0.1 M sodium citrate tribasic dihydrate (pH 5.8), 0.54 M magnesium formate dihydrate, and 10% (vol/vol) 1,2-butanediol as an additive reagent. The g5Rp-InsP_6_ complexes were prepared by mixing g5Rp with InsP_6_ at a stoichiometric ratio of 1:3. Then, using the hanging-drop vapor diffusion method, crystals of the complexes were grown from 1 M imidazole (pH 7.0) at 16°C. All crystals were transferred into solutions containing 20% (vol/vol) glycerol prior to being frozen and stored in liquid nitrogen.

### Data collection, processing, and structure determination.

The single-wavelength anomalous dispersion (SAD) data were collected using synchrotron radiation of an 0.98-Å wavelength under cryogenic conditions (100 K) at the BL18U1 beamline, Shanghai Synchrotron Radiation Facility. All diffraction data sets including g5Rp-WT and the complex with InsP_6_ were indexed, integrated, and scaled by using the HKL-2000 package (56). The selenium atoms in the asymmetric unit of SeMet-g5Rp were located and refined, and the SAD data phases were calculated and substantially improved through solvent flattening with the PHENIX program (57). A model was built manually into the modified experimental electron density using the model-building tool Coot (58) and then further refined in PHENIX. The model geometry was verified using the program MolProbity (59). Molecular replacement was used to solve the structure of the g5Rp-InsP_6_ complex, using Phaser in the CCP4 program suite with an initial search model of SeMet-g5Rp (60). Structural figures were drawn using PyMOL (DeLano Scientific). The data collection and refinement statistics are shown in Table 1.

### Surface plasmon resonance analysis.

The SPR analyses were carried out using the Biacore 8K system with a streptavidin-coated (SA) chip (catalog no. BR-1005-30; GE Healthcare) at 16°C. To reduce effects attributed to mass transport, low levels of biotin-labeled ssRNA (5′-GCUUUGAUUUCGUGCAUCUAUGGAGC-3′ and 5′-GCUUUGAUUUCG-3′) ligands (given in relative units [RU]) were immobilized on the SA chip. Given the apparent variation in RU of immobilized ligands used in different binding studies (61, 62), the average RU of immobilized 26-mer RNA and 12-mer RNA in this study are approximately 200 to 400 RU. The blank channel served as a negative control. Protein solutions with various concentrations are run across the chip at a rate of 30 μL/min and are then dissociated by running buffer (20 mM HEPES, 150 mM NaCl, 3 mM EDTA, and 0.05% Tween 20 [pH 7.5]) for 300 s at a flow rate of 30 μL/min. Regeneration of the sensor chips was performed for 30 s using regeneration buffer (0.5% SDS). The data on the binding of the g5Rp molecules to ssRNAs were fitted to a kinematic binding model, which determines association and dissociation constants by fitting the experimental data to a Langmuir model with 1:1 interaction model between analyte A and ligand B: the association (*k_a_*) and dissociation (*k_d_*) rate constants and the affinity value (*K_D_* = *k_d_*/*k_a_*) were determined.

### Microscale thermophoresis.

The binding between g5Rp and InsP_6_ was measured by microscale thermophoresis. In brief, g5Rp was first labeled using the Monolith NT protein labeling kit RED-NHS (RED fluorescent dye NT-647-NHS) (NanoTemper Technologies), and the labeled protein was then diluted to 20 nM with buffer containing 50 mM HEPES, 300 mM NaCl, and 0.05% (vol/vol) Tween 20 (pH 7.0). Then, a series of concentrations of InsP_6_ diluted in a buffer composed of 50 mM HEPES, 300 mM NaCl, and 0.05% (vol/vol) Tween 20 (pH 7.0) was added. The mixtures were loaded into capillaries and measured at ambient temperature by using 20% LED (light-emitting diode) and medium MST power in a Monolith NT.115 system (NanoTemper Technologies). The data were analyzed using NanoTemper analysis software (v.1.2.101).

### Electrophoretic mobility shift assay.

EMSAs were performed to determine the nucleic acid affinity of the wild-type and g5Rp mutants. The single-stranded nucleic acids used were 6‐carboxyfluorescein‐labeled ssRNA (5′-GCUUUGAUUUCGUGCAUCUAUGGAGC-3′) and 6‐carboxyfluorescein‐labeled ssRNA (5′-GCUUUGAUUUCG-3′) (Sheng Gong, Shanghai, China). Initially, 0.25 μM ssRNA was incubated on ice for 30 min together with different concentrations of wild type in a buffer composed of 20 mM HEPES, 60 mM KCl, 0.5 mM EDTA, 0.1% Triton X-100, 4 mM dithiothreitol (DTT), 2 mM MgCl_2_, and 5% (vol/vol) glycerol (pH 7.9); the experimental concentration of mutant g5Rp was 2 μM. To determine the effect of InsP_6_ on nucleic acid binding ability of g5Rp, we also used the EMSA to test the nucleic acid binding ability of the enzyme mixed with different concentrations of InsP_6_ (0.03 mM, 0.06 mM, 0.12 mM, 0.25 mM, 0.5 mM, 1 mM, 1.50 mM, and 2.00 mM). All samples were incubated on ice for 30 min and then electrophoresed on 4.0% native PAGE gels for 45 min at a voltage of 100 V. The results were determined with a Bio-Rad ChemiDoc MP imaging system (Bio-Rad, USA).

### Capping of the mRNA body.

For decapping assays with g5Rp, uncapped RNA was transcribed using the T7 RNA transcription kit (Vazyme) with the linear DNA (5′-CATTATTGGCCTGAAAAAGATGATTGACAGCTATAATGATTACTACAACAACGAAGTTTTCGTTAAACATAAAAACC-3′) as a template. The RNAs were capped in a 50-μL reaction system typically containing 0.4 nmol RNA, 12 μL 3,000 Ci/mmol [α-^32^P]GTP, 0.67 mM *S*-adenosylmethionine, 50 mM Tris-HCl (pH 7.6), 2 mM MgCl_2_, 6 mM KCl, 1 mM DTT, and 75 U vaccinia capping enzyme (NEB) at 37°C for 2 h. Cap-labeled RNAs were then separated from free [α-^32^P]GTP nucleotide by chromatography through a G-50 column (Sigma).

### mRNA-decapping assays.

All decapping experiments were carried out in a buffer containing 50 mM Tris-HCl (pH 7.0), 1 mM DTT, and 2 mM MnCl_2_. Generally, 100 ng of wild type or g5Rp mutants was used in each 5-μL reaction mixture. To determine the correlation between InsP_6_ and the decapping ability of g5Rp, we also tested the decapping ability of the enzyme mixed with different concentrations of InsP_6_ (0.03 mM, 0.06 mM, 0.12 mM, 0.25 mM, 0.5 mM, 1 mM, 2 mM, and 4 mM). The decapping reaction was carried out at 37°C for 60 min and then stopped with 25 mM EDTA. The products of the reaction were separated by PEI-cellulose thin-layer chromatography developed in 0.45 M (NH_4_)_2_SO_4_ and detected with autoradiography.

### Cross-linking assay.

A cross-linking assay was carried out by incubating 1 mg/mL of wild-type g5Rp or each g5Rp truncation variant and mutant I84A/I116A/L200A/I206A/F222A in a buffer containing 20 mM HEPES, 200 mM NaCl, 1 mM dithiothreitol, and 10% (vol/vol) glycerol (pH 7.5) and different concentrations of ethylene glycol *bis*(succinimidyl succinate) (EGS) for 15 min at 4°C; EGS was dissolved in 100% dimethyl sulfoxide (DMSO), and its reservoir concentration was 25 mM. The reaction was terminated by adding glycine at a final concentration of 0.15 M. The reaction products were separated by 12% SDS-PAGE and detected by Coomassie brilliant blue staining.

### Western blotting and analysis.

Two hundred thousand cells per well were seeded in a 6-well plate (Nest) and cultured for 24 h. Then, plasmids (2 μg/well) were transfected into cells using Lipofectamine 3000 (Invitrogen). After 48 h, cells were lysed in radioimmunoprecipitation assay (RIPA) buffer (Biosharp) containing protease inhibitor cocktail (MedChemExpress) and quantified by the bicinchoninic acid (BCA) kit (Sangon). About 20 μg protein was loaded in 12% SDS-PAGE gels and transferred to a polyvinylidene difluoride (PVDF) (0.22 μm) membrane (Millipore). Membranes were incubated with primary antibodies including anti-β-actin (1:10,000 dilution; HuaBio) and anti-Flag (1:1,000 dilution; CST) at 4°C overnight. After the membranes were washed three times for 10 min with Tris-buffered saline with Tween 20 (TBST) and incubated with secondary antibodies for 2 h at room temperature, they were washed three times again in TBST. Then, the blots were detected with enhanced chemiluminescence reagents (Millipore) using MiniChemi (Sagecreation, China)

### RNA extraction and quantitative real-time PCR.

Briefly, 100,000 cells were seeded in a 12-well plate (Nest) and cultured for 24 h before transfection with plasmid (1 μg/well). Total RNA was extracted using RNAiso reagent (TaKaRa), and 500 ng of total RNA was reverse transcribed using the PrimeScript RT reagent kit (TaKaRa). Subsequently, real-time PCR amplification was performed with SYBR Premix ExTag (TaKaRa) on a QuantStudio 3 system (Applied Biosystems). The cycle settings were as follows: 95°C for 30 s and then 40 cycles of 95°C for 5 s and 60°C for 34 s. Relative mRNA levels were determined using the threshold cycle (2^−ΔΔ^*^CT^*) (ΔΔ*C_T_* = Δ*C_T_* [test] – Δ*C_T_* [calibrator]) method.

### Data availability.

The structure factors and atomic coordinates of apo-g5Rp and g5Rp-InsP6 have been deposited in the Protein Data Bank under the PDB ID codes 7DNT and 7DNU, respectively.
